# Large-scale parallelization of nanomechanical mass spectrometry with weakly-coupled resonators

**DOI:** 10.1038/s41467-019-11647-2

**Published:** 2019-09-09

**Authors:** Stefano Stassi, Giulia De Laurentis, Debadi Chakraborty, Katarzyna Bejtka, Angelica Chiodoni, John E. Sader, Carlo Ricciardi

**Affiliations:** 1 Department of Applied Science and Technology, Politecnico di Torino, Corso Duca Degli Abruzzi, 24, 10129 Torino, Italy; 2 ARC Centre of Excellence in Exciton Science, School of Mathematics and Statistics, The University of Melbourne, Victoria, 3010 Australia; 3 Center for Sustainable Future Technologies, Istituto Italiano di Tecnologia, Environment Park, Building B2, Via Livorno 60, 10144 Torino, Italy

**Keywords:** NEMS, Sensors, Characterization and analytical techniques

## Abstract

Nanomechanical mass spectrometry is a recent technological breakthrough that enables the real-time analysis of single molecules. In contraposition to its extreme mass sensitivity is a limited capture cross-section that can hinder measurements in a practical setting. Here we show that weak-coupling between devices in resonator arrays can be used in nanomechanical mass spectrometry to parallelize the measurement. This coupling gives rise to asymmetric amplitude peaks in the vibrational response of a single nanomechanical resonator of the array, which coincide with the natural frequencies of all other resonators in the same array. A rigorous theoretical model is derived that explains the physical mechanisms and describes the practical features of this parallelization. We demonstrate the significance of this parallelization through inertial imaging of analytes adsorbed to all resonators of an array, with the possibility of simultaneously detecting resonators placed at distances a hundred times larger than their own physical size.

## Abstract (web summary)

Designing large-scale parallelization of nanomechanical array measurements remains elusive. Here, the authors propose weak-coupling between similar devices to evaluate the resonance frequencies of a whole resonator array with a single measurement.

## Introduction

Mass sensing using micro and nanomechanical resonators has been extensively employed in the field of chemical and biological analysis, with continuous improvements in sensitivity and throughput^1–7^. The sensitivity of resonant sensors has been enhanced to reach zeptogram resolution^8^, by scaling down the sensor dimensions to nanometer scales; yoctogram resolution has been achieved using 1D nanomaterials, such as single carbon nanotubes^9^. This extreme sensitivity has led to the development of nanomechanical mass spectrometry using resonant sensors with applications in the detection of nanoparticles and in proteomics^10–12^. New theoretical methodologies have also been developed to extract—from the adsorption-induced multi-mode eigenfrequency variation—an inertial imaging of soft/compliant adsorbates^13,14^, or the mass, position and stiffness of hard/non-compliant analytes^15,16^. In contrast to their extreme mass sensitivity, miniaturization simultaneously reduces the capture cross-section of these resonant sensors and necessitates the use of more advanced detection systems, which can hinder implementation in a practical setting^1,12^. To overcome these challenges, effort is being expended in the development of sensor arrays that inevitably require more complex readout systems to concurrently detect all sensors^10,17–19^.

Arrays of coupled micromechanical resonators have been implemented previously to parallelize detection^20^. Strong elastically coupled resonators of similar dimensions give rise to collective modes of vibration, with non-localized eigenstates, that differ significantly from isolated resonators^21^. Adsorption of an analyte on one resonator strongly perturbs the system, localizing the collective mode to a single resonator^21–24^. This Anderson or mode localization induces a strong variation of the relative amplitude of the coupled modes—not just a shift in resonance frequency—which can be used to determine both the mass and position of the analyte. Femtogram resolution using two nanomechanical coupled devices has been demonstrated^25^ and a large array of 15 microcantilevers has been implemented^26^. If more than one resonator or all resonators are perturbed simultaneously, the complexity of mode localization strongly increases and eigenmode analysis of individual resonators can become difficult to interpret.

In contrast, the vibrational modes of an array of weakly coupled resonators exhibit minor asymmetric interference peaks (commonly referred to as Fano resonances) in the vibrational response of a single resonator. These asymmetric peaks coincide with the eigenfrequencies of all other (individual) resonators of the array^27^, with each eigenfrequency being well approximated by the result for the resonator in isolation. This indicates that the response of all resonators in an array—due to adsorption on any resonator—could be monitored by measuring just a single resonator.

In this article, we show that asymmetric peaks due to weak elastic coupling can be used to parallelize the detection of analytes deposited on different nanomechanical resonators of an array—by monitoring the vibrational response of a single resonator of the same array. By employing this approach with multi-mode detection, we demonstrate simultaneous inertial imaging of multiple adsorbed masses deposited on several resonators of the array. Moreover, we establish the possibility of simultaneously detecting the resonant behavior of sensors at distances a hundred times larger than their own physical size. The present implementation of this weak-coupling detection paves the way to an unprecedentedly large-scale parallelization of nanomechanical array measurements, overcoming an (at present) challenging reduction of analysis time in these systems.

## Results

### Experimental evaluation of weak-coupling phenomenon

Elastic coupling in nanomechanical resonator arrays can be induced when nearly identical resonators are physically connected, with their vibrational response being perturbed by neighboring resonators. The type of physical connection affects the strength of coupling. Strongly coupled cantilever resonators are often realized by connecting the resonators via a suspended overhang (ledge) of similar thickness to the cantilevers themselves. This gives rise to collective modes of vibration that differ considerably from a single device. In contrast, when the resonators are connected via a stiff support, e.g., a bulk substrate, elastic coupling between the resonators can be small. In such weak elastic coupling, the resonators are observed to exhibit a (primary) Lorentzian vibrational amplitude response that is well approximated by that of a single resonator, plus asymmetric peaks positioned in the frequency domain at eigenfrequencies (if the resonators were considered in isolation) of all other weakly coupled resonators of the array^27^. The amplitudes of these asymmetric coupling peaks are small—often several orders of magnitude smaller than the primary Lorentzian resonance peak—which can hinder their detection relative to the primary peak. Moreover, these asymmetric amplitudes are found to depend on the distance between the resonators and on the shape of connecting bulk substrate. In this work, we use arrays of nominally identical microcantilevers (see Methods section) where weak elastic coupling is induced by the bulk substrate that connects the resonators; this substrate has a slope of 54.7° relative to the cantilevers, due to the KOH etching process. No suspended overhang (ledge) connects the resonators—eliminating the possibility of strong coupling behavior and thus strongly entangled eigenstates. In previous work, we observed weak-coupling peaks in commercial resonators arrays procured from IBM (Concentris, Type CLA-500-070-04V2)—which are (intrinsically) fabricated to minimize coupling between similar resonators^27^. Weak-coupling is always present when there is a physical (not perfectly rigid) connection between the resonators. Since the amplitude of the coupling peaks can be very small in these situations, their presence can be easily masked.

**Fig. 1:**
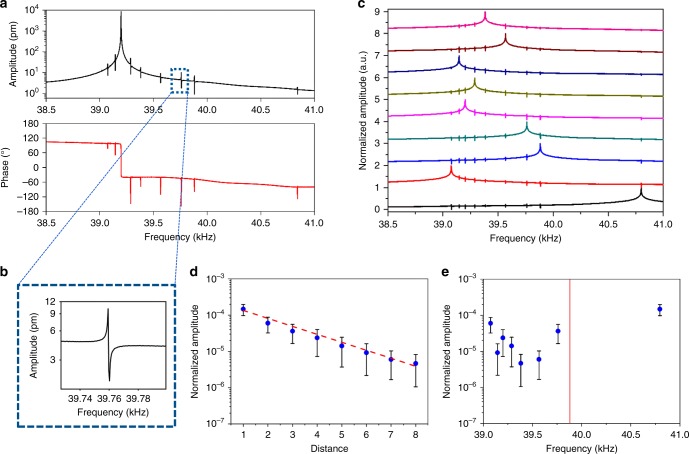
Measured weak-coupling in a nanomechanical cantilever array. **a** Amplitude and phase response of a single cantilever, showing the asymmetric weak-coupling peaks which are related to the other resonators of the array; see subfigure (**c**). **b** Magnified view of the asymmetric amplitude peak contained in the dotted box of subfigure (**a**). **c** Amplitude response of all nine cantilever resonators of the array, showing coincidence of the weak-coupling peaks with primary resonances of other cantilevers. Vibration spectra are normalized and shifted in amplitude for clarity. **d** Average peak-to-peak amplitudes of all weak-coupling asymmetric peaks as a function of the corresponding distance (of the cantilever related to that peak) to the cantilever being measured; line of best fit shown. **e** Average peak-to-peak amplitudes of the weak-coupling peaks as a function of frequency; no correlation is evident. Measurements on each cantilever are reported in Supplementary Table 1

The device array used initially in this study consists of nine cantilevers that are spaced 170 µm apart (see Methods). The sub-picometer resolution of the Laser Doppler Vibrometer (LDV) measurement (see Methods), allows detection of the asymmetric weak-coupling peaks, whose peak-to-peak amplitudes are several orders of magnitude smaller than the amplitude of the primary Lorentzian resonance peak. All amplitude spectra are reported on a logarithmic scale. Figure 1a gives the measured asymmetric weak-coupling peaks in both the amplitude and phase spectra, which are acquired on a single microcantilever of the array; an expanded scale showing the asymmetric weak-coupling peak is provided in Fig. 1b. Figure 1c shows that the frequency of each asymmetric peak coincides with the primary Lorentzian resonances of all other cantilevers in the array. The dimensions and material of each cantilever resonator are nominally identical yet small differences in the observed resonance frequencies inevitably arise due the fabrication process.

The asymmetric weak-coupling peaks are studied in detail to understand their dependence on parameters of the array. First, the coupling phenomenon is examined as function of distance between the resonators. The peak-to-peak (i.e., minimum to maximum) amplitude of each asymmetric amplitude peak is measured as a function of physical position to the corresponding cantilever with the primary Lorentzian resonance (at the same frequency). This is performed for the measured amplitude response of each cantilever in the array, which results in nine different spectra with eight asymmetric weak-coupling peaks each. All the individual measurements report the same trend: the amplitudes of the asymmetric weak-coupling peaks generally decrease with distance to the associated primary resonator with a Lorentzian response at the same frequency (Supplementary Fig. 1). Averaging this dataset shows that these peak-to-peak amplitudes of the weak-coupling peaks decrease as a function of above-mentioned distance; see Fig. 1d. The fitted curve displays near exponential decay with distance. We note that these amplitudes are not expected to exactly coincide with distance because coupling between resonators will also depend on their relative stiffnesses; this is explored further below. Finally, the peak-to-peak amplitudes of the weak-coupling peaks are certainly uncorrelated with frequency; see Fig. 1e and Supplementary Fig. 2.

**Fig. 2:**
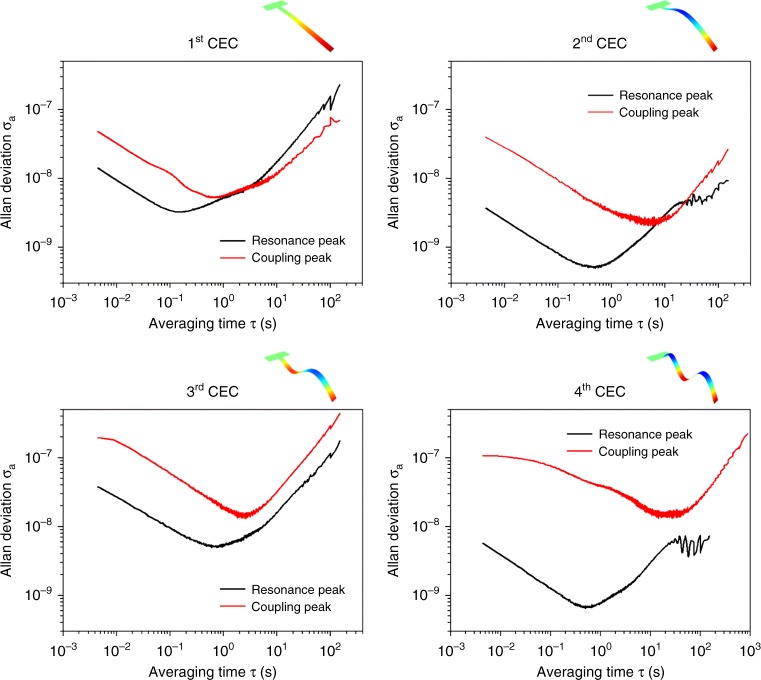
Frequency stability of Lorentzian resonance and weak-coupling peaks. Measured Allan deviations for the first four cantilever modes (as a function of averaging time); each of these modes is referred to as a collective eigenmode class (CEC) below. Results are reported for the amplitude of the primary Lorentzian peak and one weak-coupling peak

Although the peak-to-peak amplitudes of the asymmetric weak-coupling peaks are small relative to the primary resonance peaks, the reported measurements show that they are easily detected with good signal-to-noise. Allan deviations of the measured frequencies of both primary and weak-coupling peaks are determined, results of which are given in Fig. 2. As expected, the (low amplitude) weak-coupling peaks exhibit higher frequency noise and require longer averaging times to reach the (lower) values of the primary resonances. Nonetheless, Allan deviations of all peaks are <0.1 part per million (ppm) for averaging times between 0.01 and 1 s. These averaging times are sufficient to use the weak-coupling peaks of the first four resonance modes for mass spectroscopy; each of these four modes is referred to as a collective eigenmode class (CEC) below.

### Theoretical model

A theoretical model is formulated for an arbitrary array of *N* near-identical cantilevers; the shape and properties of the cantilevers are arbitrary, as is the substrate connecting the cantilevers. Rather than considering each cantilever in isolation, the collective modes of the cantilever array are calculated numerically—allowing coupling between the cantilevers to be rigorously specified. This is most readily achieved using a commercial finite element solver, such as COMSOL Multiphysics (used here). The array is assumed to operate in vacuum with constant damping; a good approximation in the limit of small damping. As in the reported measurements, the array is excited by oscillating its clamping structure, i.e., the substrate. Eigenfunction decomposition is used to formulate the solution.

The collective eigenmodes of the cantilever array are solved using full 3D linear elastic simulations, in the absence of damping (which is included later). Because the *N* cantilevers are nearly identical, yet all different, the array exhibits *N* distinct collective eigenmodes for every eigenmode of an isolated cantilever; each one of these sets of *N* distinct collective eigenmodes is henceforth referred to as a collective eigenmode class (CEC). Cantilevers exhibit a monotonic increase in deflection from the base to the free end for the first (fundamental) CEC; higher-order CECs contains nodes, see Fig. 2. For each distinct collective eigenmode of the array, one cantilever displays large amplitude while the others exhibit small amplitudes (due to weak-coupling); these small amplitudes vanish in the limit of zero coupling, i.e., an infinitely rigid substrate. Due to the absence of damping, cantilevers vibrating with small amplitude will either be in-phase or 180° out-of-phase with the cantilever that vibrates at large amplitude.

We construct an *N* × *N* matrix, **A**, whose *n*th column consists of the calculated vertical displacement, $z_m^{(n)}$, of cantilevers *m* = 1,2,…,*N* (order along cantilever array) at a fixed (laser spot) position for the *n*th collective eigenmode of the cantilever array, i.e.,

1 $${\mathbf{A}} = \left( {z_m^{(n)}} \right),m,n = 1,2, \ldots ,N,$$

where the index *m* refers to the matrix row, with entries normalized such that the maximum displacement is unity (for the cantilever with the largest displacement). The displacement vector, **w**^(*n*)^, in the frequency domain relative to the clamping substrate, for the *n*th collective eigenmode of the array (including damping), whose rows are the displacements of cantilevers 1, 2, ..., *N*, is then

2 $${\mathbf{w}}^{(n)} = a_{{\mathrm{drive}}}\frac{{\omega ^2 + \frac{{i\omega \omega _n}}{{Q_n}}}}{{\omega _n^2 - \omega ^2 - \frac{{i\omega \omega _n}}{{Q_n}}}}{\mathbf{A}} \cdot {\mathbf{b}}^{\left( n \right)},$$

with the unitary vector,

3 $${\mathbf{b}}^{(n)} = \left( {\delta _{mn}} \right),m = 1,2, \ldots ,N,$$

where *δ*_*mn*_ is the Kronecker delta function, *i* is the imaginary unit, *ω* is angular frequency, *ω*_*n*_ is the resonant frequency of the *n*th collective eigenmode, *Q*_*n*_ is its corresponding quality factor and *a*_drive_ is a constant for each CEC that is proportional to the oscillation amplitude applied to the clamping structure of the cantilever array; an implicit time dependence of exp(−*iωt*) is assumed, where *t* is time.

The complete displacement frequency response relative to the clamping structure, for a given CEC, is then obtained by superposing the result obtained in Eq. (2) over all collective eigenmodes, giving

4 $${\mathbf{w}} = \mathop {\sum}\nolimits_{n = 1}^N {{\mathbf{w}}^{(n)}} = a_{{\mathrm{drive}}}\mathop {\sum}\nolimits_{n = 1}^N {\frac{{\omega ^2 + \frac{{i\omega \omega _n}}{{Q_n}}}}{{\omega _n^2 - \omega ^2 - \frac{{i\omega \omega _n}}{{Q_n}}}}{\mathbf{A}} \cdot {\mathbf{b}}^{\left( n \right)},}$$

where the *m*^th^ row of **w** contains the response of cantilever *m*. The complex-valued solution in Eq. (4) contains the entire response of the cantilever array system, which can be compared directly with the measured amplitude and phase responses.

### Influence of quality factor on weak-coupling peaks

We now study the experimental conditions that enable the weak-coupling peaks to be observed in the amplitude response. Two cases are considered separately and then combined.

*Case 1*: First, we consider when the frequency of the weak-coupling peak lies outside of the full-width-half-maximum (FWHM) of the primary Lorentzian peak, which is controlled by the *Q*-factor, i.e., $\left| {\omega _n - \omega _s} \right| \gg \omega _s/Q_s$, where *ω*_*n*_ is the frequency of the weak-coupling peak under consideration while *ω*_*s*_ and *Q*_*s*_ are the resonant frequency and quality factor of the primary Lorentzian resonance, respectively; because the cantilevers are nearly identical, a high *Q*-factor is required. The complete displacement vs. frequency response of cantilever *s* in this case is given by the *s*th row of **w** in Eq. (4), i.e.,

5 $$w_s \approx a_{{\mathrm{drive}}}\left( {\frac{{\omega ^2 + \frac{{i\omega \omega _s}}{{Q_s}}}}{{\omega _s^2 - \omega ^2}} + \mathop {\sum}\nolimits_{\begin{array}{*{20}{c}} {n = 1} \\ {\left( {n \ne s} \right)} \end{array}}^N {\frac{{\omega ^2 + \frac{{i\omega \omega _n}}{{Q_n}}}}{{\omega _n^2 - \omega ^2 - \frac{{i\omega \omega _n}}{{Q_n}}}}z_n^{\left( s \right)}} } \right).$$

For the weak-coupling peak that originates from neighboring cantilever *n*, i.e., at *ω* = *ω*_*n*_, to be measured in the response of (primary) cantilever *s*, we require the *n*th term in the sum of Eq. (5) to be significant relative to the first term at *ω* = *ω*_*n*_, i.e.,

6 $$Q_n\left| {z_n^{\left( s \right)}} \right| \gtrsim A_{{\mathrm{min}}}\left| {\frac{{\omega _n^2}}{{\omega _s^2 - \omega _n^2}}} \right|,$$

where the ‘minimum relative amplitude factor’, *A*_min_, is (typically) an order one constant that depends on specifics of the experimental setup, i.e., its signal-to-noise ratio; see below. For example, *A*_min_ = 3 corresponds to a minimum weak-coupling peak amplitude that is three times that of the primary Lorentzian peak tail at *ω* = *ω*_*n*_. Because all cantilevers are nearly identical, Eq. (6) is well approximated by

7 $$\left| {z_n^{\left( s \right)}\left( {1 - \frac{{\omega _n}}{{\omega _s}}} \right)} \right| \gtrsim \frac{{A_{{\mathrm{min}}}}}{{2Q_n}}.$$

Equation (7) is satisfied only if the *Q*-factor is very large because the coupling is weak, i.e., $z_n^{\left( s \right)}$ is small, and 1−*ω*_*n*_/*ω*_*s*_ is also small.

*Case 2*: Next, we consider when the weak-coupling peak overlaps with the primary Lorentzian resonance peak, i.e., the *Q*-factor or frequency spacing between the peaks are sufficiently small. Equation (5) then establishes that the weak-coupling peak is measurable only if

8 $$\left| {z_n^{\left( s \right)}} \right| \gtrsim A_{{\mathrm{min}}},$$

which can violate the weak-coupling assumption, i.e., $| {z_n^{\left( s \right)}} | \ll 1,$ because *A*_min_ is typically order one. Therefore, a weak-coupling peak cannot be observed if it overlaps with the primary resonance, unless a detection system with exquisite signal-to-noise and precision is used.

Combining the above formulas then gives the required inequality that must be satisfied for the weak-coupling peak of cantilever *n* to be observed in the displacement response of cantilever *s*,

9 $$\frac{{A_{{\mathrm{min}}}}}{{\left| {z_n^{\left( s \right)}} \right|}} \lesssim \max \left( {1,2Q_n\left| {1 - \frac{{\omega _n}}{{\omega _s}}} \right|} \right)$$

Equation (9) shows that the $Q$-factor can control visibility of the weak-coupling peaks; in addition to the coupling strength between resonators as defined by $z_n^{\left( s \right)}$ and the detection system sensitivity. Indeed, Eq. (9) can be used to determine the lowest permissible *Q*-factor for the weak-coupling peaks to be detectable, using a single measurement of the cantilever array at high *Q*. This is because $z_n^{\left( s \right)}$, *ω*_*n*_, and *ω*_*s*_ are controlled by the array geometry and material properties, and hence are independent of dissipation, i.e., *Q*_*n*_. For any array, $| {z_n^{\left( s \right)}} |$ can be determined by measuring the ratio of maximum and minimum amplitudes of the asymmetric weak-coupling peak (Fig. 1b) henceforth denoted as *α*_ratio_ (>1), and using:

10 $$\left| {z_n^{\left( s \right)}} \right| = \left| {\frac{{\alpha _{{\mathrm{ratio}}} - 1}}{{2Q_n\sqrt {\alpha _{{\mathrm{ratio}}}} \left( {1 - \frac{{\omega _n}}{{\omega _s}}} \right)}}} \right|.$$

This is derived from Eq. (5) in the limit of large *Q*_*n*_ using the property that the resonators are nearly identical; the amplitude ratio, *α*_ratio_, will vary with *Q*_*n*_ for a given array. The corresponding expression for *α*_ratio_ is

11 $$\alpha _{{\mathrm{ratio}}} = 1 + 2\beta ^2\left( {1 + \sqrt {1 + \frac{1}{{\beta ^2}}} } \right),$$

where $\beta \equiv Q_nz_n^{\left( s \right)}\left( {1 - \frac{{\omega _n}}{{\omega _s}}} \right)$, which is a monotonically increasing function of *β*. This shows that increasing the *Q*-factor or coupling strength as defined by $z_n^{\left( s \right)}$ enhances the presence (amplitude) of the weak-coupling peaks.

### Features of the theoretical model

Before presenting theoretical results for the frequency response, we highlight some generic features observed from finite element analysis of the 9-cantilever array:

*Feature 1*: Reordering the columns of the **A**-matrix, so that its main diagonal contains the largest magnitude entries (unity), gives a matrix that is well approximated by the identity matrix plus an antisymmetric matrix. This approximation is found to systematically improve as the coupling strength, $z_n^{\left( s \right)}$, decreases; the error in this approximation is ~10% for the cantilever array studied in Fig. 1 (Supplementary Fig. 4). Since the columns of this reordered matrix give the individual cantilever displacements for each collective eigenmode, $z_m^{(n)}$, this finding shows that the coupling strength between each *cantilever pair* of the array is fixed by their distance. Importantly, the coupling strength between two cantilevers is expected to not only depend on distance but also on the relative difference between the stiffnesses of the cantilevers. This is evidenced by the reordered matrix not being a Toeplitz (diagonal-constant) matrix, i.e., it exhibits strong variations along each diagonal (Supplementary Tables 3–6).

*Feature 2:* The collective (displacement) eigenmodes display the following general property: individual cantilevers of higher stiffness than the primary cantilever vibrate in phase with the primary cantilever, while those of lower stiffness are 180° out-of-phase. This is expected because the primary cantilever’s natural frequency (if it were isolated) is higher than that of the softer ones (and thus above their individual resonant frequencies)—while the primary cantilever’s natural frequency is lower than the stiffer ones (and thus below their individual resonant frequencies). Such a discussion is relevant in the weak-coupling limit only (as assumed) and ignores cantilevers at the ends of the array, i.e., end effects. This feature explains existence of the antisymmetric coupling matrix mentioned above.

The finding that the reordered **A**-matrix is the sum of the identity matrix and an antisymmetric matrix can be used to experimentally test the theoretical model. The magnitude of its entries, $| {z_m^{\left( n \right)}} |$, can be measured from the ratio of the maximum and minimum amplitudes of the (asymmetric) weak-coupling peaks; Eq. (10). The matrix constructed from $| {z_m^{\left( n \right)}} |$, denoted the **A**’-matrix, is expected to be approximately symmetric (since the reordered **A**-matrix contains an antisymmetric matrix) and this theoretical prediction can be compared with experimental measurement.

The measured data reported in Fig. 1c are analyzed in this fashion, the results of which are reported in Supplementary Table 2. This shows that the measured values of $| {z_m^{\left( n \right)}} |$ vary by two orders-of-magnitude; a numerical factor of 150 times. Even so, the relative error between the entries of the **A**′-matrix, which are $| {z_m^{\left( n \right)}} |$, and its transpose is small; it varies between 1 and 63%, with a mean of 32% (Supplementary Table 3 and Supplementary Fig. 3). This difference increases with increasing frequency difference between the primary Lorentzian peak and the weak-coupling peak, and decreasing magnitude of $| {z_m^{\left( n \right)}} |$. This is consistent with the above theoretical considerations and the **A**′-matrix data obtained using the theoretical model (Supplementary Tables 4–6). Experimental uncertainty also increases **A**′ asymmetry. The signal-to-noise ratio reduces as $| {z_m^{\left( n \right)}} |$ decreases, which is consistent with the observed increase in asymmetry in **A**′-matrix with decreasing $| {z_m^{\left( n \right)}} |$. Importantly, the measured dataset in Supplementary Table 2 shows that the reordered matrix, the **A**′-matrix, is not a Toeplitz (diagonally constant) matrix, highlighting that distance between the cantilevers is not the only factor controlling their coupling. Thus, even though amplitudes of the weak-coupling peaks are strongly affected by distance between the cantilevers (Fig. 1d), the relative stiffness between the individual cantilevers must also have a significant effect.

**Fig. 3:**
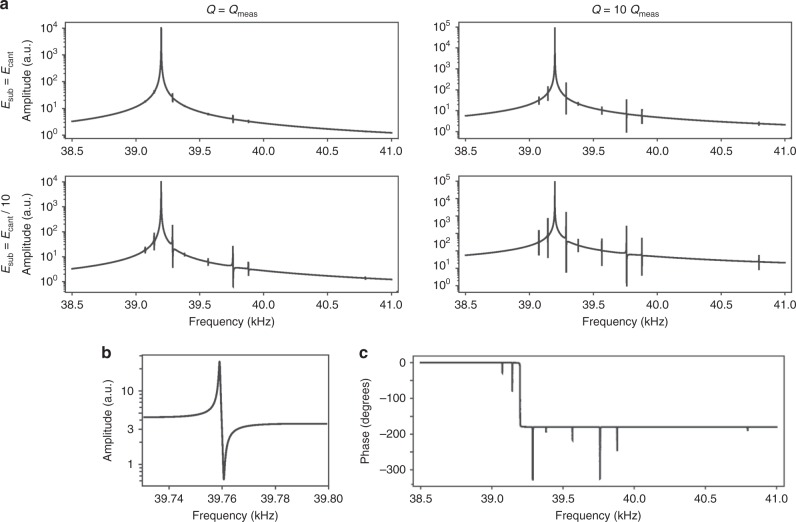
Theoretical model of weak-coupling in a mechanical resonator array. **a** Amplitude vs. frequency response of the 9-cantilever array calculated using the theoretical model. The first (fundamental) CEC is studied, where cantilever deflection increases monotonically from the base to the free end. Results given as a function of substrate elasticity (relative to the cantilever elastic modulus) and quality factor. Reducing substrate elasticity and increasing the $Q$-factor enhances the weak-coupling peaks, which is consistent with Eq. (9). **b** Weak-coupling peak centered at 39.76 kHz, for substrate modulus 10 times smaller than cantilevers; compare with Fig. 1a. **c** Phase response for substrate modulus 10 times smaller than cantilever; compare with Fig. 1a

Figure 3a gives results obtained using the theoretical model for the amplitude vs. frequency response of the cantilever (of the 9-cantilever array) reported in Fig. 1a. These results are presented as a function of the *Q*-factor and substrate elasticity (to vary the coupling strength). The measured *Q*-factor of this cantilever array is ~80,000 and varies slightly between each cantilever (Supplementary Table 1). These slightly different *Q*-factors for each cantilever are used in the theoretical model. Figure 3a shows that if the substrate has the same elastic modulus as the cantilevers, the weak-coupling peaks do exist but are of small amplitude relative to measurements in Fig. 1a. Reducing the substrate Young’s modulus by a factor of 10 strongly enhances the weak-coupling peaks and gives results that resemble the measurements of Fig. 1a. The measured asymmetric weak-coupling peak in Fig. 1b is also well-reproduced by the theoretical model (Fig. 3b). Importantly, the 9-cantilever array is formed by bonding a silicon wafer onto a silica covered substrate (silicon-on-insulator SOI wafer). Comparison of Figs 1a and 3a thus strongly suggests that this bonding is not rigid and displays finite elasticity. This must enhance the coupling between the cantilevers and amplify the weak-coupling peaks. Increasing the *Q*-factor (see Fig. 3a) also leads to an enhancement in the amplitudes of the weak-coupling peaks, as predicted above. Since the bonding material between the cantilever and silica layer of SOI substrate is difficult to characterize and model, we refrain from making a direct quantitative comparison of theory to measurement. Even so, we note that the measured coupling strengths—as dictated by $| {z_m^{\left( n \right)}} |$—are long-ranged relative to theoretical simulations on the idealized structure (no bonding layer), see Supplementary Tables 2, 4–6; this may be due to the presence of the bonding layer in measurements and its absence in simulations.

These findings show that it is important to not use a highly rigid substrate that may suppress coupling beyond the detection limit of instrumentation. It also shows that a reduction in dissipation (enhancement in *Q*-factor) is also desirable. While these effects can compete, the measurements reported in Fig. 1 confirm that such operation is entirely practical. Knowledge of these competing requirements will thus enable optimization of future sensor arrays.

Figure 3c provides the complementary phase response for a substrate modulus that is 10 times smaller than the cantilevers. This is to be compared with the measured phase response in Fig. 1a, which bears a striking resemblance. There are some subtle differences. First, the phase drops immediately across the primary Lorentzian response (at 39.2 kHz) is measured to be 140°, which differs from the theoretically expected and observed 180°. Second, some (not all) weak-coupling phase peaks in measurement are asymmetric in structure, while theory predicts a symmetric phase response (Supplementary Figs 7–9 and Supplementary Note 1). Both these differences vary between different (nominally identical) fabricated cantilever arrays and are not always present (Supplementary Figs 7–9), suggesting that they are driven by non-idealities in fabrication and transduction.

### Parallelization of frequency measurements for inertial imaging

The above weak-coupling phenomenon can be used to strongly reduce the measurement times of resonator arrays. Measurement of similar resonators can be parallelized by observing the amplitude spectrum of a single resonator—which will contain a primary Lorentzian peak and weak-coupling peaks. A single measurement on a single cantilever is all that is needed to detect the responses of all cantilevers over the entire resonator array. We illustrate the utility of this approach by conducting inertial imaging^14^ experiments on individual adsorbates.

**Fig. 4:**
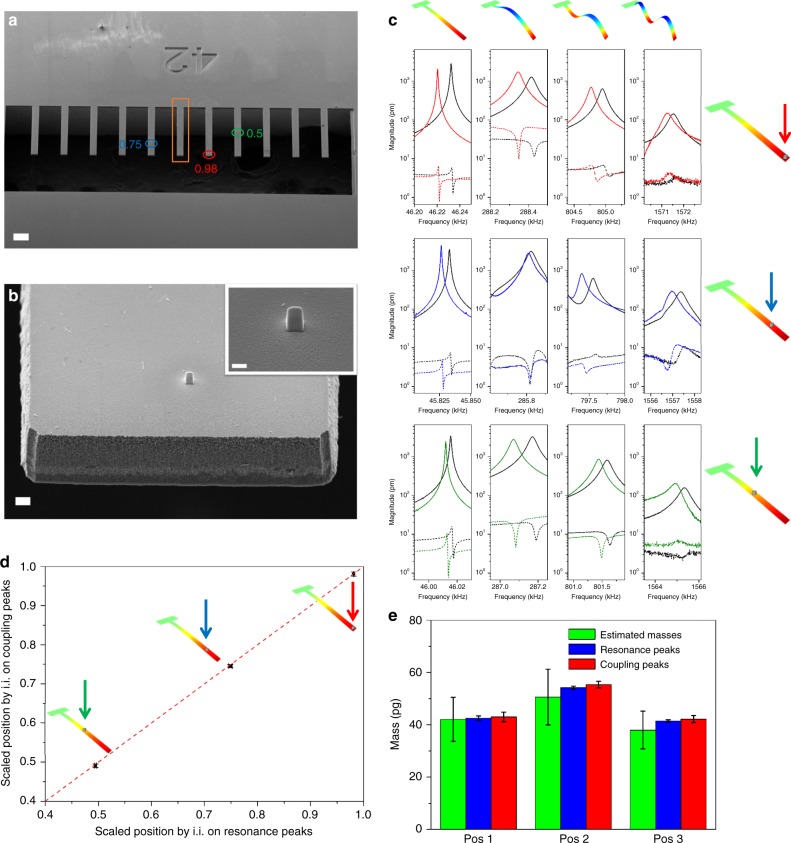
Inertial imaging of adsorbed masses. **a** SEM image of the resonator array used. The positions of the three deposited masses are underlined with an elliptical sign, while the cantilever in the box is the resonator used for the weak-coupling peak measurement. The scale bar is 100 µm. **b** Details of one of the deposited mass. The scale bars are 2 µm in the image and 1 µm in the inset. **c** Comparison of the resonance peaks and coupling peaks for the first four CECs. Solid curves are the vibrational responses of the resonators with the deposited masses (Pos1 in the top graph, Pos2 in the center, and Pos3 in the bottom), while the dotted line is the response of the resonator in the central position of the array. **d** Position of the deposited mass determined with inertial imaging using the primary Lorentzian peak shifts and the average weak-coupling peak shifts. The error bars are determined by the fitting residuals and frequency noise. **e** Masses estimated by SEM and with inertial imaging from the primary Lorentzian and weak-coupling peak shifts

Separate masses are deposited on three cantilevers of an 11-cantilever array (Fig. 4a, b). The masses are added using focus ion beam (FIB) induced deposition of platinum to precisely control the position and quantity of deposited material. The masses are placed at three different positions over the cantilever lengths: Pos1 = 0.98 (7th cantilever of the array from the left), Pos2 = 0.75 (5th cantilever), Pos3 = 0.5 (8th cantilever), scaled by the cantilever length and are the relative distances from the clamp. The weak-coupling peaks are used to inertially image the test masses in a highly parallel fashion. We employ the recent theoretical methodology for inertial imaging proposed by Sader et al.^13^, that uses frequency data from multi-mode measurements of the resonator, to simultaneously determine the mass, position and higher-order central moments of the adsorbate mass (details in Supplementary Note 2).

For each cantilever with a deposited mass, the first four CECs are recorded before and after deposition to measure the frequency shifts induce by deposition. In addition, the vibrational spectra of all the other cantilevers of the array are recorded to measure the frequency shifts via the weak-coupling peaks. Figure 4c reports the primary Lorentzian resonances and the weak-coupling peaks for the first four CECs, and for the three different mass positions (Pos1, Pos2, and Pos3). The weak-coupling peaks are recorded on the central cantilever of the array (6th cantilever of the array, box in Fig. 4a), while the average frequency of the weak-coupling peaks of all cantilevers are used for inertial imaging. From the relative frequency shifts of the different CECs, we inertial image the three mass configurations and determine the mass, relative position and variance.

Separate analyses using the primary Lorentzian resonance peaks and the weak-coupling peaks, show excellent agreement for the measured position (Fig. 4d and Table 1) and mass (Fig. 4e) of the adsorbates. The reported (small) uncertainty in measurements is calculated from the fitted residual and frequency noise.

**Table 1:** Mass, position and variance of the deposited mass evaluated by SEM and calculated with the inertial imaging approach using (primary) Lorentzian resonance and weak-coupling peak shifts

Sample	Position 1	Position 2	Position 3
Mass (SEM) (pg)	42.07 (±20%)	50.59 (±21%)	37.94 (±19%)
Mass (Lorentzian) (pg)	42.51 (±2.05%)	54.14 (±1.17%)	48.65 (±1.17%)
Mass (weak-coupling) (pg)	42.98 (±4.26%)	55.32 (±2.3%)	42.16 (±3.15%)
Position (SEM)	0.98	0.75	0.5
Position (Lorentzian)	0.981 (±0.05%)	0.749 (±0.43%)	0.494 (±0.41%)
Position (weak-coupling)	0.980 (±0.55%)	0.745 (±0.51%)	0.491 (±0.88%)
Variance (SEM)	1.18 × 10^−5^	1.11 × 10^−5^	8.09 x 10^−6^
Variance (Lorentzian)	9.38 × 10^−6^ (±5.2%)	1.44 × 10^−5^ (±7.6%)	9.67 × 10^−7^ (±32%)
Variance (weak-coupling)	1.78 × 10^−5^ (±36.1%)	1.32 × 10^−5^ (±10.8%)	1.125 × 10^−6^ (±29.1%)

SEM reported mass uses measured geometric dimensions and platinum density. Position 1 = 0.98, Position 2 = 0.75, Position 3 = 0.5, scaled relative to the cantilever length and relative to the clamp

Table 1 shows that good agreement is also observed for variances of the masses, obtained using the weak-coupling peaks and primary Lorentzian peaks. The inertial imaging results are comparable to those measured using SEM analysis, while some differences exist. These differences are likely related to the stiffnesses of the deposited masses^2^. Current inertial imaging methodologies are formulated for soft and compliant adsorbate^13,14^, while the use of stiff adsorbates can lead to error in the high central moments, e.g., variance, skewness of the adsorbate.

The resolution of inertial imaging based on multi-mode measurements is limited by the modal frequency noise of the resonator, rather than any finite wavelength effect^14^. Frequency fluctuations (specified by Allan deviations) for the first four CECs are used to determine the minimum resolvable feature size of an adsorbate. For the 460-µm long cantilevers used in this work, a near nanometer scale spatial resolution is predicted, both with primary Lorentzian peaks (S.D. of adsorbate density distribution of 35 nm) and coupling peaks (S.D. of 120 nm). This confirms the possibility of using the weak-coupling peaks to measure mass and position—with nanometer scale resolution—for each analyte on any cantilever of the array, simply with a single measurement of the frequency response of a single resonator. The use of weak-coupling peaks can enable significant parallelization of the measurement, leading to strong reduction of measurement time.

**Fig. 5:**
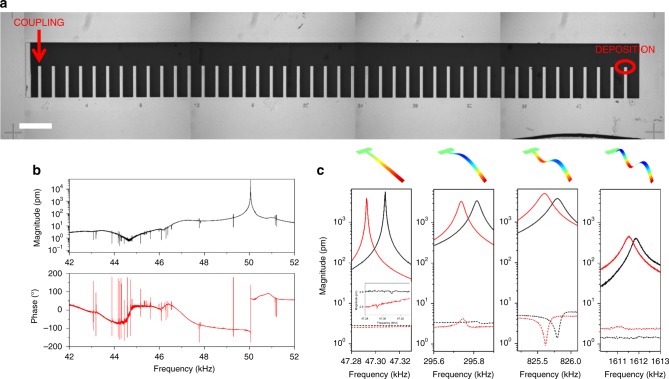
Weak-coupling peak analysis on a large 44-resonator array. **a** SEM image of the resonator array composed by 44 nominally identical cantilevers. Platinum mass is deposited on the right-most resonator, while the weak-coupling peaks are measured on the left-most resonator. The scale bar is 500 µm. **b** Amplitude and phase of the vibration spectrum of the left-most cantilever of the array showing the asymmetric weak-coupling peaks which are related to all other 43 resonators. **c** Comparison of the primary Lorentzian and weak-coupling peaks for the first four CECs. Solid curves are the resonator responses with the deposited mass placed in the right-most position, while the dotted curves are the response of the resonator in the left-most position of the array

To demonstrate the efficacy of weak-coupling peak approach for large-scale parallelization, an array of 44 cantilevers is fabricated and characterized (Fig. 5a). The measured vibrational spectrum of one cantilever of the array (Fig. 5b) shows 43 weak-coupling peaks, in addition to the primary Lorentzian resonance of the cantilever. These weak-coupling peaks have a frequency and vibrational amplitude that depends on the physical distance between the cantilevers and their stiffness. Larger the distance, generally the smaller the amplitude of these weak-coupling peaks, as already reported in Fig. 1d. Similar behavior is also observed in the phase signal.

A test platinum mass is deposited using FIB on the end tip of the right-most cantilever; see Fig. 5a and Supplementary Fig. 10. The estimated mass and relative position (obtained using SEM) are 46.1 ± 12.1 pg and 0.96, respectively. The frequency shifts induced by this added mass are clearly evident in the first four CECs (solid curves in Fig. 5c). Weak-coupling peaks related to this resonator are recorded by measuring the vibration spectrum of the left-most cantilever. Even if the amplitudes of these weak-coupling peaks are small, they can still be detected above the noise level (dashed curves in Fig. 5c). They produce a similar frequency shift to that of the right-most cantilever (where the platinum mass is deposited). In this device, since the physical distance between the left- and right-most resonators is 9 mm (50 μm cantilever width and 150 μm resonator spacing), this shows that the weak-coupling effect can propagate over distances more than 170 times the lateral size of the single resonator. Both the fractional frequency shifts measured from the primary Lorentzian resonance and from the weak-coupling peaks are again used for inertial imaging of the deposited adsorbate. Comparison of the determined values for the mass (47.7 and 52.2 pg with primary and weak-coupling peaks, respectively) and for the relative position (0.962 and 0.952, respectively) of the platinum deposit agree; variance measurements are not performed due to poor signal-to-noise; the 4th CEC peaks are barely visible. This confirms the feasibility of the proposed coupled resonator readout for large resonator arrays.

### Limit of detection

**Fig. 6:**
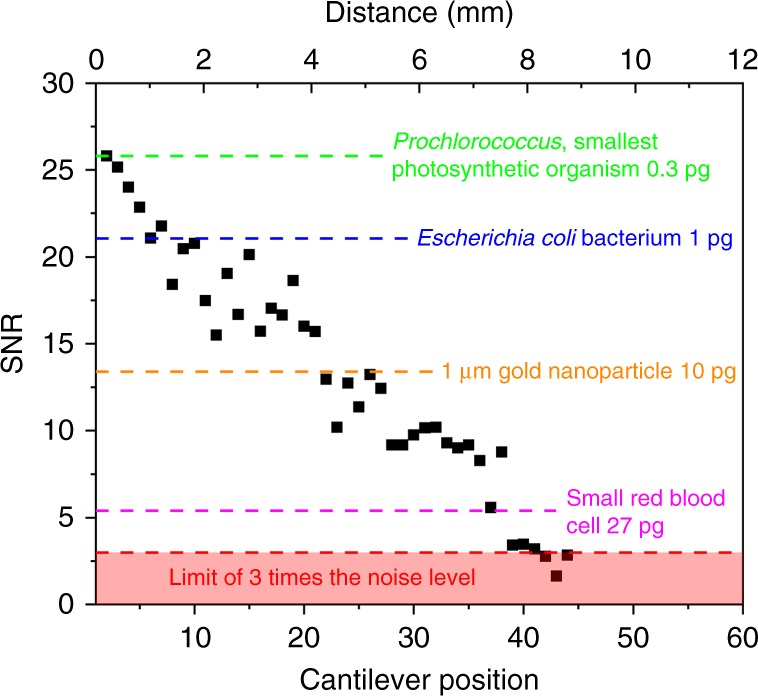
Minimum detectable mass for weak-coupling peaks. Signal-to-noise ratio (SNR) of the weak-coupling peaks of a 44 resonators array as a function of the position from the cantilever under measurement. The physical distance is also reported on the top axis. The limit of detection for the weak-coupling peaks is reported with dashed lines corresponding to the mass of some typical (nominated) targets of interest in chemical and biological sensing

The feasibility of parallelizing the readout of many resonators over large distances is demonstrated above. Since the amplitudes of the weak-coupling peaks decrease with distance, the minimum detectable mass varies with the distance too. On the other hand, mass responsivity remains unchanged because it depends on the physical dimensions of the resonator (around 70 Hz ng^−1^ for the cantilevers under analysis). To quantify the variation of this limit of detection (LOD), the signal-to-noise ratio (SNR) of each weak-coupling peak (first CEC) is measured. Figure 6 shows that the SNR decreases linearly with distance between the measurement cantilever and the source cantilever that drives the weak-coupling peak. A SNR level of 3 is reached around the 41th resonator position (from the left), which may represent a LOD. This SNR level is chosen because it is the standard limit in spectroscopy to confirm the existence of a resonance peak, and in the statistics of biological assays to verify the presence of a data point (three times the standard deviation)^28–31^. The weak-coupling peaks for cantilevers in the 41th to the 44th positions are still observable (see Fig. 5c), but in principle they would not be suitable for sensing due to their poor SNR. The reported theoretical LOD, or minimum detectable mass for each resonator, is obtained using:

12 $${\mathrm{LOD}} \equiv - 2M\frac{{\Delta f_{{\mathrm{noise}}}}}{{f_r}},$$

where *M* is the mass of the resonator and *f*_*r*_ is the frequency of the weak-coupling peak. The minimum detectable frequency shift, $\Delta f_{{\mathrm{noise}}}$, associated with the weak-coupling peak is defined as the resonance peak width at an amplitude of *A*_max_−3*A*_noise_, where *A*_max_ and *A*_noise_ are the maximum amplitude of the resonance peak and the noise amplitude, respectively (Supplementary Fig. 11). Details regarding calculation of the SNR and LOD are reported in Supplementary Note 3. For the array under consideration, the minimum detectable mass varies from 0.3 pg for the 2nd resonator from the left, corresponding to the mass of the smallest photosynthetic organism, up to ~ 30 pg in the 40th resonator, i.e., the average mass of a red blood cell.

## Discussion

We have demonstrated that the detection of weak-coupling resonance peaks among nearly identical resonators in a single array can be used to parallelize measurement of the entire array. A commensurate theory was derived that explains the dominant features of the detection scheme. Increasing the resonator coupling through a reduction in substrate elasticity and/or increasing the quality factors of the resonators were shown to be advantageous. By monitoring the weak-coupling peaks, we demonstrated the possibility of obtaining an inertial image—with nanometer resolution—of a mass adsorbed onto each resonator of the array, by just monitoring one resonator. Moreover, we showed it is possible to simultaneously detect the resonances of structures separated by distances that are hundreds of times larger than their physical size. We envision that this proposed weak-coupling readout can bring to an unprecedentedly high parallelization of detection, leading to a strong reduction in analysis time.

## Methods

### Fabrication of the resonator arrays

The resonator arrays are fabricated using standard microfabrication techniques described elsewhere^17^. The arrays of 9 resonators (590-μm long, 70-μm wide, and 7-μm thick) and 11 and 44 resonators (460-μm long, 50-μm wide, and 7-μm thick) consist of nominally identical silicon microcantilevers connected to the bulk substrate which presents a slope of 54.7° due to potassium hydroxide etching. The spacing between each cantilever resonator is 170 μm in the 9-resonator array and 150 μm in the 11 and 44 resonator ones.

### Mass addition by FIB induced deposition

Focused Ion Beam (FIB) system (Dual Beam Auriga, Zeiss) is used to deposit platinum, on the cantilevers, in a controlled fashion in terms of the position and the quantity. The gas precursor for platinum deposition is methylcyclopentadienyl-trimethylplatinum (CH_3_)_3_Pt(CpCH_3_) and the ion-induced deposition is carried out at 30 kV with the current of 10 pA, and the beam is scanned over a 1 × 1 μm^2^ area. Morphological analyses of the deposited masses are carried out with field emission scanning electron microscope (FESEM, Dual Beam Auriga, Zeiss). Material deposited by FIB results amorphous and deposition with similar conditions results in a platinum density of ~12 ± 1.2 g cm^−3 ^^32^. This density and the dimensions (measured from SEM images) are used to estimate the masses deposited.

### Characterization of the resonator vibrational response

The vibrational response of the mechanical resonators is measured using a LDV (MSA-500, Polytec Gmbh); a single point measurement is performed on each cantilever of the array. The resonator arrays are mounted with an adhesive tape on a piezoelectric disk used for actuation. All the measurements are performed at a vacuum level of 2 × 10^−7^ mbar in a chamber evacuated by a membrane and a turbomolecular pumps (MINI-Task System, Varian Inc. Vacuum Technologies). The vibrational spectra were recorded actuating the piezodisk with a sinusoidal chirp signal generated by the LDV system, in the specific frequency range of interest.

The frequency stabilities of the resonators are evaluated by computing the Allan deviation, *σ*_*a*_, of the primary Lorentzian peak and the center frequency of the weak-coupling peaks, in the integration time *τ*:

$$\sigma _a = \sqrt {\frac{1}{{2(N_a - 1)}}\mathop {\sum }\limits_{i = 2}^N \left( {\frac{{\bar f_i - \bar f_{i - 1}}}{{f_0}}} \right)^2,}$$

where $\bar f_i$ is the time average of the frequency measurement in the *i*th time interval of duration *τ*, *N*_*a*_ is the total number of time intervals, and *f*_0_ is the mean resonance frequency over the duration of the measurement. The Allan deviation measurement is performed using a lock-in system (HF2LI, Zurich Instruments).

### Finite element analysis

The **A**-matrix in Eq. (1) is evaluated using 3D finite element simulations (COMSOL Multiphysics) of the complete array structure, ignoring damping. This involves solving Navier’s equation with traction free boundary condition on all free surfaces, except for the bottom horizontal surface of the substrate that is fixed. The Young’s modulus of the substrate and cantilever is specified separately and a Poisson’s ratio of 0.3 is used throughout. The mesh is systematically refined to achieve convergence of better than 99%.

## Supplementary information

Supplementary Information

Peer Review File

## Data Availability

The data that support the findings of this study are available from the corresponding authors upon reasonable request.
