# Supplementary material for: Large-scale parallelization of nanomechanical mass spectrometry with weakly-coupled resonators
Source: Nat Commun. 2019 Sep 9;10:3647. doi: 10.1038/s41467-019-11647-2 (PMC6733932; doi:10.1038/s41467-019-11647-2)
Supplement: Supplementary file 1 — Supplementary Information [file 41467_2019_11647_MOESM1_ESM.pdf]

SUPPLEMENTARY INFORMATION

Large-scale parallelization of nanomechanical mass spectrometry with weakly-coupled resonators

Stefano Stassi[a], Giulia De Laurentis[a], Debadi Chakraborty[b], Katarzyna Bejtka[c], Angelica Chiodoni[c], John E. Sader[b,*], Carlo Ricciardi[a,*]

[a] *Department of Applied Science and Technology, Politecnico di Torino, Corso Duca Degli Abruzzi, 24, 10129 Torino, Italy*

[b] *ARC Centre of Excellence in Exciton Science, School of Mathematics and Statistics, The University of Melbourne, Victoria 3010, Australia*

[c] *Center for Sustainable Future Technologies, Istituto Italiano di Tecnologia, Environment Park, Building B2, Via Livorno 60, 10144 Torino, Italy*

[*]*Corresponding Authors: jsader@unimelb.edu.au, carlo.ricciardi@polito.it*

SUPPLEMENTARY INFORMATION LIST

Supplementary Table 1. Resonant frequencies and quality factors of the primary Lorentzian peaks

Supplementary Figure 1. Physical distance dependence of weak coupling peak-to-peak amplitude

Supplementary Figure 2. Frequency distance dependence of weak coupling peak-to-peak amplitude

Supplementary Table 2. Experimental $\mathbf{A}'$-matrix of 9-cantilever array

Supplementary Table 3. Percentage relative difference between the $\mathbf{A}'$-matrix and its transpose

Supplementary Figure 3. Percentage degree of asymmetry in experimentally $\mathbf{A}'$-matrix

Supplementary Table 4. Theoretical $\mathbf{A}'$-matrix of 9-cantilever array for $E_{\mathrm{substrate}} = E_{\mathrm{cantilever}}$

Supplementary Figure 4. Percentage degree of asymmetry in $\mathbf{A}'$-matrix for $E_{\mathrm{substrate}} = E_{\mathrm{cantilever}}$

Supplementary Table 5. Theoretical $\mathbf{A}'$-matrix of 9-cantilever array for $E_{\mathrm{substrate}} = E_{\mathrm{cantilever}}/10$

Supplementary Figure 5. Percentage degree of asymmetry in $\mathbf{A}'$-matrix for $E_{\mathrm{substrate}} = E_{\mathrm{cantilever}}/10$

Supplementary Table 6. Theoretical $\mathbf{A}'$-matrix of 9-cantilever array for $E_{\mathrm{substrate}} = E_{\mathrm{cantilever}}/100$

Supplementary Figure 6. Percentage degree of asymmetry in $\mathbf{A}'$-matrix for $E_{\mathrm{substrate}} = E_{\mathrm{cantilever}}/100$

Supplementary Figure 7. Phase peak symmetry of a 9-cantilever array

Supplementary Figure 8. Phase peak symmetry of a 11-cantilever array

Supplementary Figure 9. Evaluation of phase peak symmetry in another 11-cantilever array

Supplementary Figure 10. Image of the mass deposited on the 44-cantilever array

Supplementary Figure 11. Scheme of the $\Delta f_{\mathrm{noise}}$ evaluation

Supplementary Notes 1. Symmetry of the weak-coupling phase peaks

Supplementary Note 2. Inertial imaging methodology

Supplementary Note 3. Signal-to-noise ratio of the 44-cantilever array

SUPPLEMENTARY FIGURES AND TABLES

Supplementary Table 1. Resonant frequencies and quality factors of the primary Lorentzian peaks. The table reports the resonant frequencies and quality factors of each resonator (left-to-right on chip) of the 9-cantilever array studied in Figure 1; for the first (fundamental) collective eigenmode class (CEC), where cantilever deflection increases monotonically from the base to the free end. Data are obtained by exciting the cantilever array and fitting the primary Lorentzian peaks.

**Res**	**1**	**2**	**3**	**4**	**5**	**6**	**7**	**8**	**9**
***f* (kHz)**	40.795	39.073	39.880	39.759	39.198	39.287	39.143	39.566	39.381
Q	57151	64742	73634	79883	86183	89187	88708	85890	82853

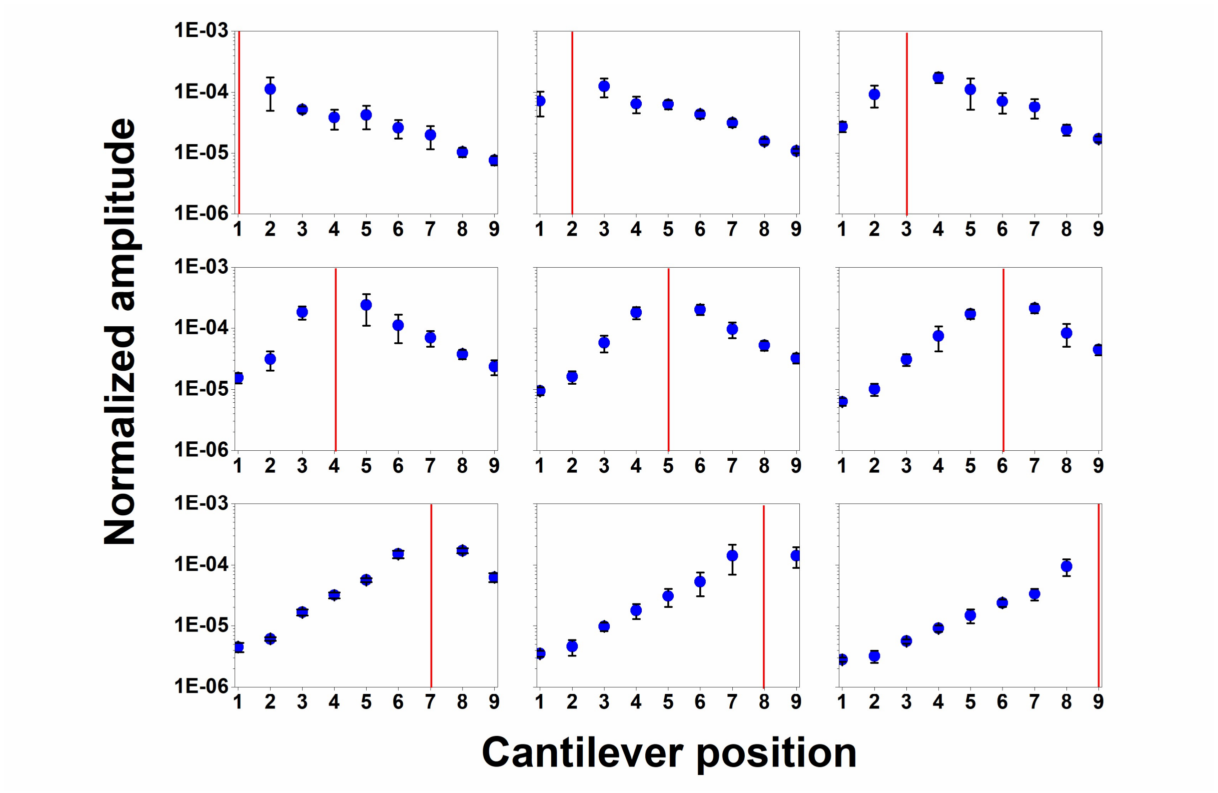

Supplementary Figure 1. Physical distance dependence of weak coupling peak-to-peak amplitude. Peak - to-peak amplitude of weak-coupling asymmetric peaks as a function of physical position of the cantilever in the array. The amplitude is normalized with respect to the primary Lorentzian resonance peak. The red line represents the position in the array of the resonator on which the measurement is performed. One graph for each resonator of the array is reported. The peak-to-peak amplitudes of the asymmetric weak-coupling peaks decrease with physical distance to the associated primary resonator (that exhibits a Lorentzian response at the same frequency).

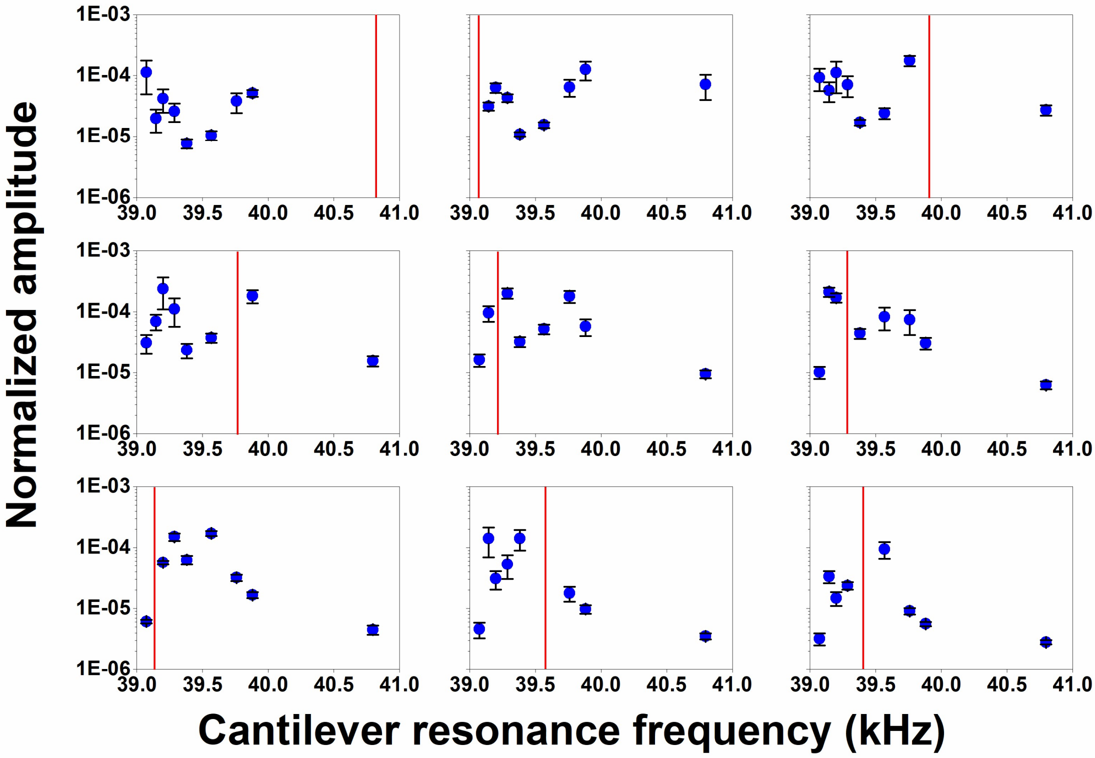

Supplementary Figure 2. Frequency distance dependence of weak coupling peak-to-peak amplitude. Peak -to-peak amplitude of weak-coupling asymmetric peaks as a function of the resonance frequency of the cantilever in the array. The amplitude is normalized with respect to the primary Lorentzian resonance peak. The red line represents the resonance frequency of the resonator on which the measurement is performed. One graph for each resonator of the array is reported. In contrast to the observed dependence of the coupling peaks with physical distance among the resonators (Supplementary Figure 1), no relationship is found in between the peak-to-peak amplitude of the weak-coupling peak and its resonance frequency.

Supplementary Table 2. Experimental $\mathbf{A}'$-matrix of 9-cantilever array. The table reports the $\mathbf{A}'$-matrix of 9-cantilever array experimentally measured using Equation (10) of the manuscript and data in Fig. 1c (diagonal entries are unity, by definition) for the first (fundamental) CEC. Absolute value of $\mathbf{A}$-matrix is denoted as the $\mathbf{A}'$-matrix and is defined: $\mathbf{A}' = (|A_{ij}|)$.

Res	1	2	3	4	5	6	7	8	9
**1**	1	0.001657	0.001916	0.001238	0.000592	0.000479	0.000342	0.000378	0.000263
**2**	0.001925	1	0.003627	0.002423	0.009132	0.004073	0.00894	0.001038	0.001218
**3**	0.002133	0.003671	1	0.030055	0.00292	0.002427	0.001382	0.002403	0.001044
**4**	0.00148	0.002828	0.027184	1	0.005856	0.004448	0.002181	0.005121	0.001751
**5**	0.000938	0.014221	0.003541	0.006324	1	0.033312	0.030574	0.003346	0.004476
**6**	0.000745	0.006578	0.00313	0.004953	0.034965	1	0.018599	0.005688	0.010969
**7**	0.000587	0.017098	0.002263	0.003006	0.038996	0.022325	1	0.006241	0.005233
**8**	0.000565	0.0017	0.00349	0.007183	0.004405	0.007411	0.007031	1	0.011976
**9**	0.00042	0.0023	0.001876	0.002953	0.007156	0.016501	0.007761	0.01533	1

Supplementary Table 3. Percentage relative difference between the $\mathbf{A}'$-matrix and its transpose. The table reports the percentage relative differences between the experimentally measured $\mathbf{A}'$-matrix of 9-cantilever array (Supplementary Table 2) and its transpose i.e., $2(\mathbf{A}'_{ij} - \mathbf{A}'^{\mathrm{T}}_{ij})/(\mathbf{A}'_{ij} + \mathbf{A}'^{\mathrm{T}}_{ij})$.

Res	1	2	3	4	5	6	7	8	9
**1**	0	-15	-11	-18	-45	-43	-53	-40	-46
**2**	15	0	-1	-15	-44	-47	-63	-48	-61
**3**	11	1	0	10	-19	-25	-48	-37	-57
**4**	18	15	-10	0	-8	-11	-32	-34	-51
**5**	45	44	19	8	0	-5	-24	-27	-46
**6**	43	47	25	11	5	0	-18	-26	-40
**7**	53	63	48	32	24	18	0	-12	-39
**8**	40	48	37	34	27	26	12	0	-25
**9**	46	61	57	51	46	40	39	25	0

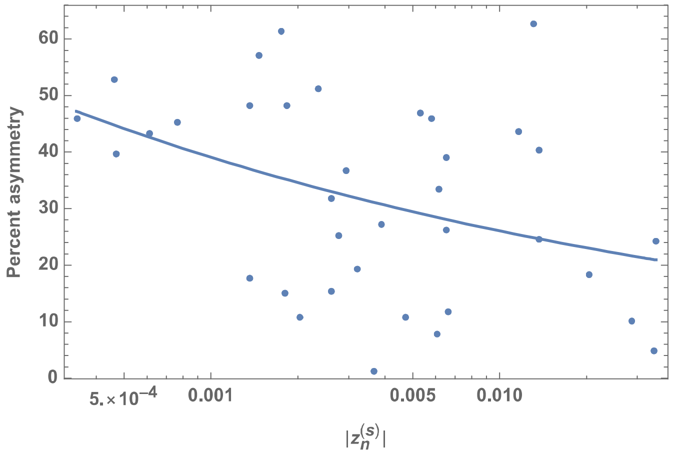

Supplementary Figure 3. Percentage degree of asymmetry in experimentally $\mathbf{A}'$-matrix. The graph reports the percentage degree of asymmetry in the experimentally $\mathbf{A}'$-matrix (Supplementary Table 3) as a function of $\left|z_n^{(s)}\right|$ – diagonal entries ignored. The percentage of asymmetry is computed as $2(A'_{ij} - A'^{T}_{ij})/(A'_{ij} + A'^{T}_{ij})$. Power-law curve best fit: $\text{percent degree} \approx 11.6/z_n^{0.18}$.

Supplementary Table 4. Theoretical $\mathbf{A}'$-matrix of 9-cantilever array for $\boldsymbol{E}_{\mathbf{substrate}} = \boldsymbol{E}_{\mathbf{cantilever}}$. The table reports the $\mathbf{A}'$-matrix of 9-cantilever array measured using Equation (10) of the manuscript and data obtained from the finite element simulation for the first (fundamental) CEC using a Young's modulus of the substrate equal to the cantilevers one. Absolute value of $\mathbf{A}$-matrix is denoted as the $\mathbf{A}'$-matrix and is defined: $\mathbf{A}' = (|A_{ij}|)$.

**Res**	**1**	**2**	**3**	**4**	**5**	**6**	**7**	**8**	**9**
**1**	1	8.3E-05	3.6E-05	1.3E-05	4E-06	1.8E-06	5.7E-07	1.4E-07	6.9E-08
**2**	7.91382E-05	1	0.00019	5.8E-05	0.00014	3.8E-05	5E-05	2.4E-06	5.3E-07
**3**	3.49071E-05	0.0002	1	0.00133	6.2E-05	3.1E-05	1.2E-05	1.1E-05	1.9E-06
**4**	1.30362E-05	5.9E-05	0.00132	1	0.00029	8.9E-05	3.1E-05	4.3E-05	7.1E-06
**5**	0.00000388	0.00014	6.1E-05	0.00028	1	0.0018	0.00076	4.8E-05	3.4E-05
**6**	0.00000175	3.9E-05	3.1E-05	8.9E-05	0.0018	1	0.00111	0.00014	0.00014
**7**	0.000000548	5E-05	1.2E-05	3E-05	0.00076	0.0011	1	0.00037	0.00013
**8**	0.000000133	2.5E-06	1.1E-05	4.3E-05	4.8E-05	0.00014	0.00037	1	0.00075
**9**	0.000000066	5.5E-07	1.8E-06	7E-06	3.4E-05	0.00014	0.00013	0.00075	1

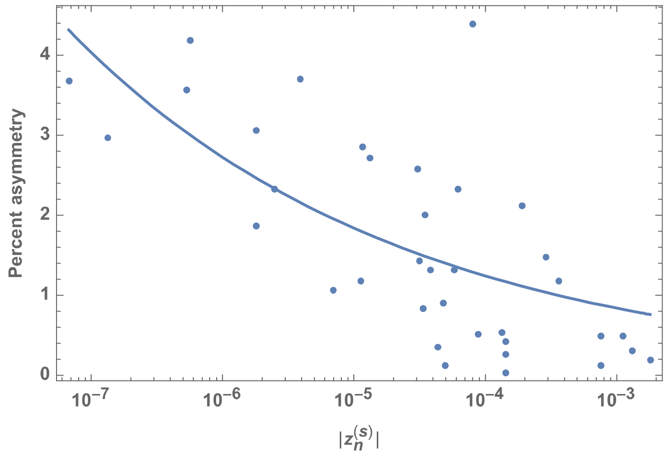

Supplementary Figure 4. Percentage degree of asymmetry in $\mathbf{A}'$-matrix for $\boldsymbol{E}_{\mathbf{substrate}} = \boldsymbol{E}_{\mathbf{cantilever}}$. The graph reports the percentage degree of asymmetry of the $\mathbf{A}'$-matrix obtained from the simulation with $E_{\text{substrate}} = E_{\text{cantilever}}$ condition (Supplementary Table 4), as a function of $\left|z_n^{(s)}\right|$ – diagonal entries ignored. The percentage of asymmetry is computed as $2(A'_{ij} - A'^{T}_{ij})/(A'_{ij} + A'^{T}_{ij})$. Power-law curve best fit: $\text{percent degree} \approx 0.26/z_n^{0.17}$.

Supplementary Table 5. Theoretical $\mathbf{A}'$-matrix of 9-cantilever array for $E_{\text{substrate}} = E_{\text{cantilever}}/10$. The table reports the $\mathbf{A}'$-matrix of 9-cantilever array measured using Equation (10) of the manuscript and data obtained from the finite element simulation for the first (fundamental) CEC using a Young's modulus of the substrate ten times lower than the cantilevers one. Absolute value of $\mathbf{A}$-matrix is denoted as the $\mathbf{A}'$-matrix and is defined: $\mathbf{A}' = (|A_{ij}|)$.

**Res**	**1**	**2**	**3**	**4**	**5**	**6**	**7**	**8**	**9**
**1**	1	0.00078	0.00031	0.00012	3.3E-05	1.4E-05	4.7E-06	1.1E-06	6.4E-07
**2**	0.00074	1	0.00178	0.00053	0.00121	0.00031	0.00045	2E-05	4E-06
**3**	0.0003	0.00182	1	0.01233	0.00054	0.00026	0.00011	9.5E-05	1.5E-05
**4**	0.00011	0.00052	0.0123	1	0.00268	0.00072	0.00028	0.00036	5.8E-05
**5**	3.2E-05	0.00121	0.0005	0.00264	1	0.0167	0.00687	0.0004	0.00028
**6**	1.4E-05	0.00034	0.00026	0.00076	0.01666	1	0.01037	0.00122	0.0012
**7**	4.5E-06	0.00044	9.4E-05	0.00025	0.00704	0.01022	1	0.0034	0.00118
**8**	1E-06	2.3E-05	9.1E-05	0.00036	0.0004	0.00119	0.00346	1	0.00707
**9**	6E-07	5.1E-06	1.4E-05	5.7E-05	0.00029	0.00118	0.00118	0.00704	1

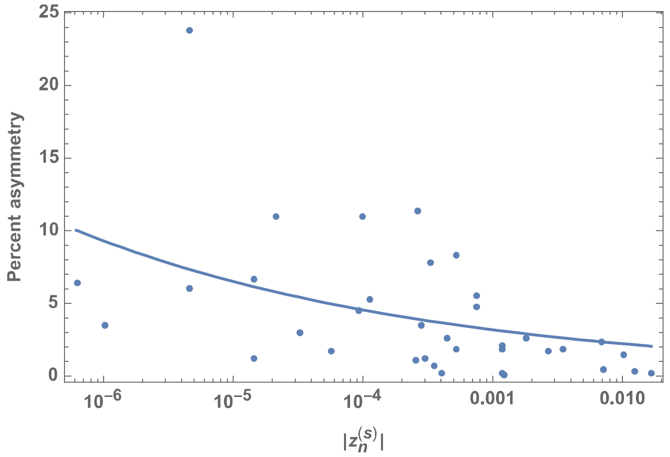

Supplementary Figure 5. Percentage degree of asymmetry in $\mathbf{A}'$-matrix for $E_{\text{substrate}} = E_{\text{cantilever}}/10$. The graph reports the percentage degree of asymmetry of the $\mathbf{A}'$-matrix obtained from the simulation with $E_{\text{substrate}} = E_{\text{cantilever}}/10$ condition (Supplementary Table 5), as a function of $\left|z_n^{(s)}\right|$ – diagonal entries ignored. The percentage of asymmetry is computed as $2(A'_{ij} - A'^{T}_{ij})/(A'_{ij} + A'^{T}_{ij})$. Power-law curve best fit: percent degree $\approx 1.09/z_n^{0.16}$.

Supplementary Table 6. Theoretical $\mathbf{A}'$-matrix of 9-cantilever array for $E_{\text{substrate}} = E_{\text{cantilever}}/100$. The table reports the $\mathbf{A}'$-matrix of 9-cantilever array measured using Equation (10) of the manuscript and data obtained from the finite element simulation for the first (fundamental) CEC using a Young's modulus of the substrate one hundred times lower than the cantilevers one. Absolute value of $\mathbf{A}$-matrix is denoted as the $\mathbf{A}'$-matrix and is defined: $\mathbf{A}' = (|A_{ij}|)$.

Res	1	2	3	4	5	6	7	8	9
**1**	1	0.00321	0.00119	0.00051	0.00014	5.4E-05	2.3E-05	7.1E-06	1.7E-06
**2**	0.003035933	1	0.00721	0.00247	0.00536	0.00122	0.00245	9.9E-05	2.7E-05
**3**	0.001208558	0.00751	1	0.05034	0.0023	0.00094	0.00053	0.00042	7.3E-05
**4**	0.000448474	0.0022	0.0502	1	0.01112	0.00226	0.00151	0.00144	0.00025
**5**	0.000136094	0.0053	0.00169	0.01088	1	0.06767	0.03182	0.00157	0.00112
**6**	6.42094E-05	0.00166	0.00096	0.00306	0.06636	1	0.04365	0.00467	0.00494
**7**	2.16195E-05	0.00221	0.00036	0.00102	0.03472	0.0414	1	0.01381	0.00522
**8**	0.00000691	0.00014	0.00036	0.00151	0.00143	0.00414	0.01439	1	0.02905
**9**	0.00000135	4.8E-05	5.9E-05	0.00024	0.00122	0.00454	0.00509	0.02898	1

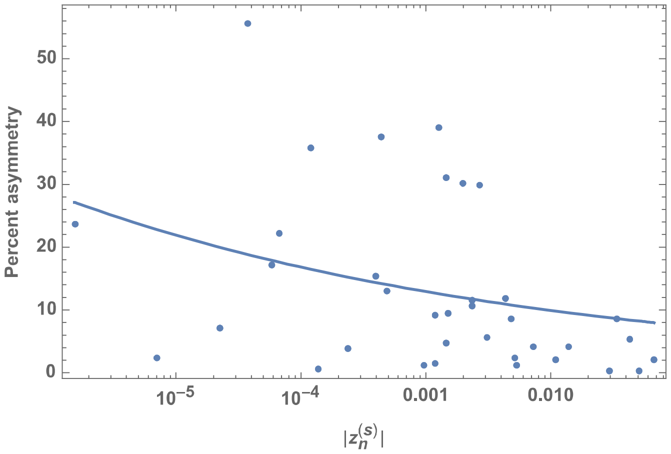

Supplementary Figure 6. Percentage degree of asymmetry in $\mathbf{A}'$-matrix for $E_{\text{substrate}} = E_{\text{cantilever}}/100$. The graph reports the percentage degree of asymmetry of the $\mathbf{A}'$-matrix obtained from the simulation with $E_{\text{substrate}} = E_{\text{cantilever}}/100$ condition (Supplementary Table 6), as a function of $\left|z_n^{(s)}\right|$ – diagonal entries ignored. The percentage of asymmetry is computed as $2(A'_{ij} - A'^{T}_{ij})/(A'_{ij} + A'^{T}_{ij})$. Power-law curve best fit: percent degree $\approx 5.85/z_n^{0.11}$.

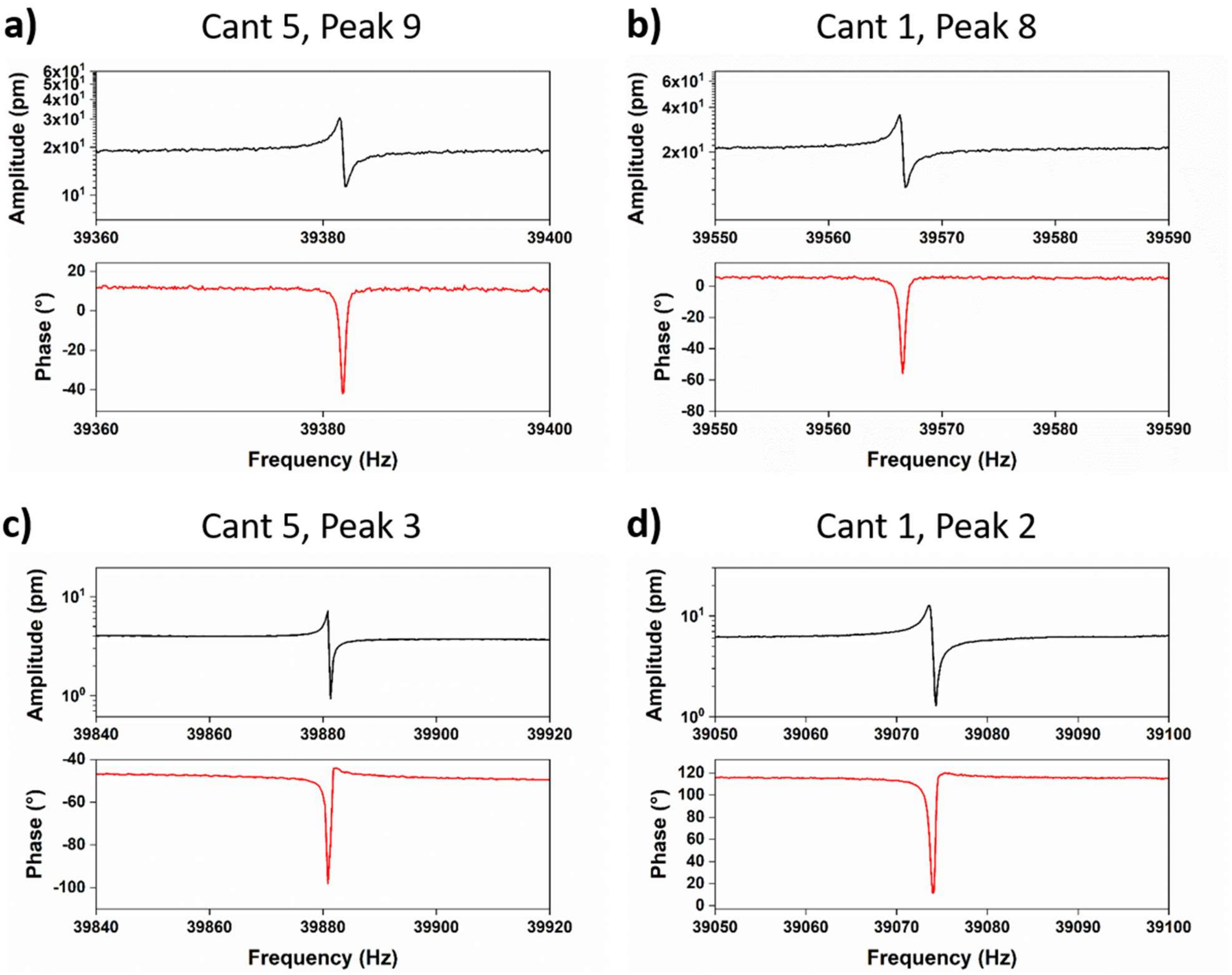

Supplementary Figure 7. Phase peak symmetry of a 9-cantilever array. Amplitude and phase response of different weak-coupling peaks of a 9-cantilever array. The device is identical to that reported in Figure 1. The cantilever and the resonance peak measured are indicated, e.g., Cant 5, Peak 9 corresponds to detection of Cantilever 5 from the left, with the weak-coupling peak corresponding to Cantilever 9 (from the left). (a,b) Phase peaks with a (near) symmetric response, (c,d), asymmetric phase responses. Lorentzian peaks of Cantilever 5 and Cantilever 1 are located at 39,198 Hz and 40,795 Hz, respectively.

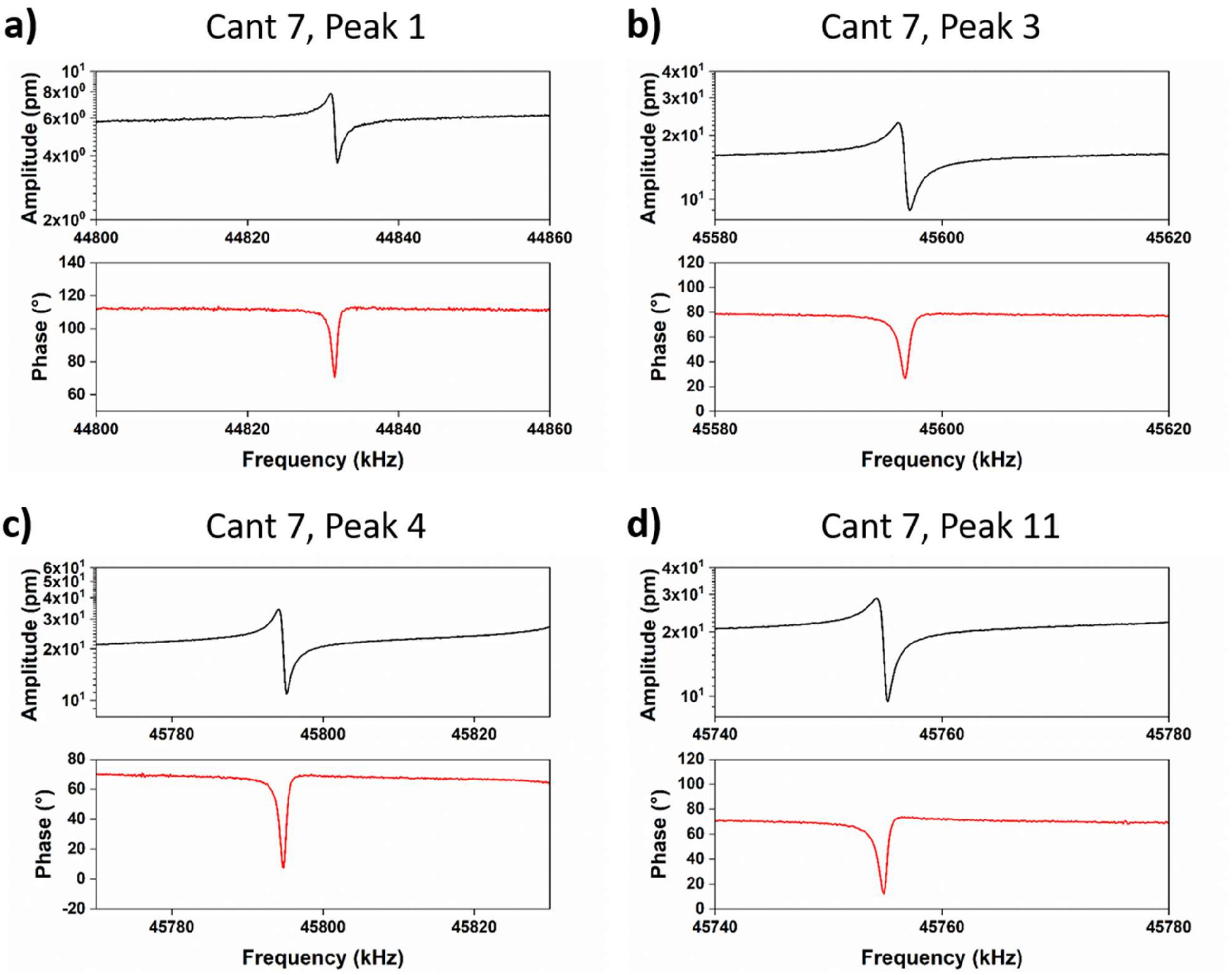

Supplementary Figure 8. Phase peak symmetry of a 11-cantilever array. Results analogous to Supplementary Figure. 7, but for an 11-cantilever array. See caption of Supplementary Figure 7 for details. Lorentzian peak of Cantilever 7 is located at 46,232 Hz.

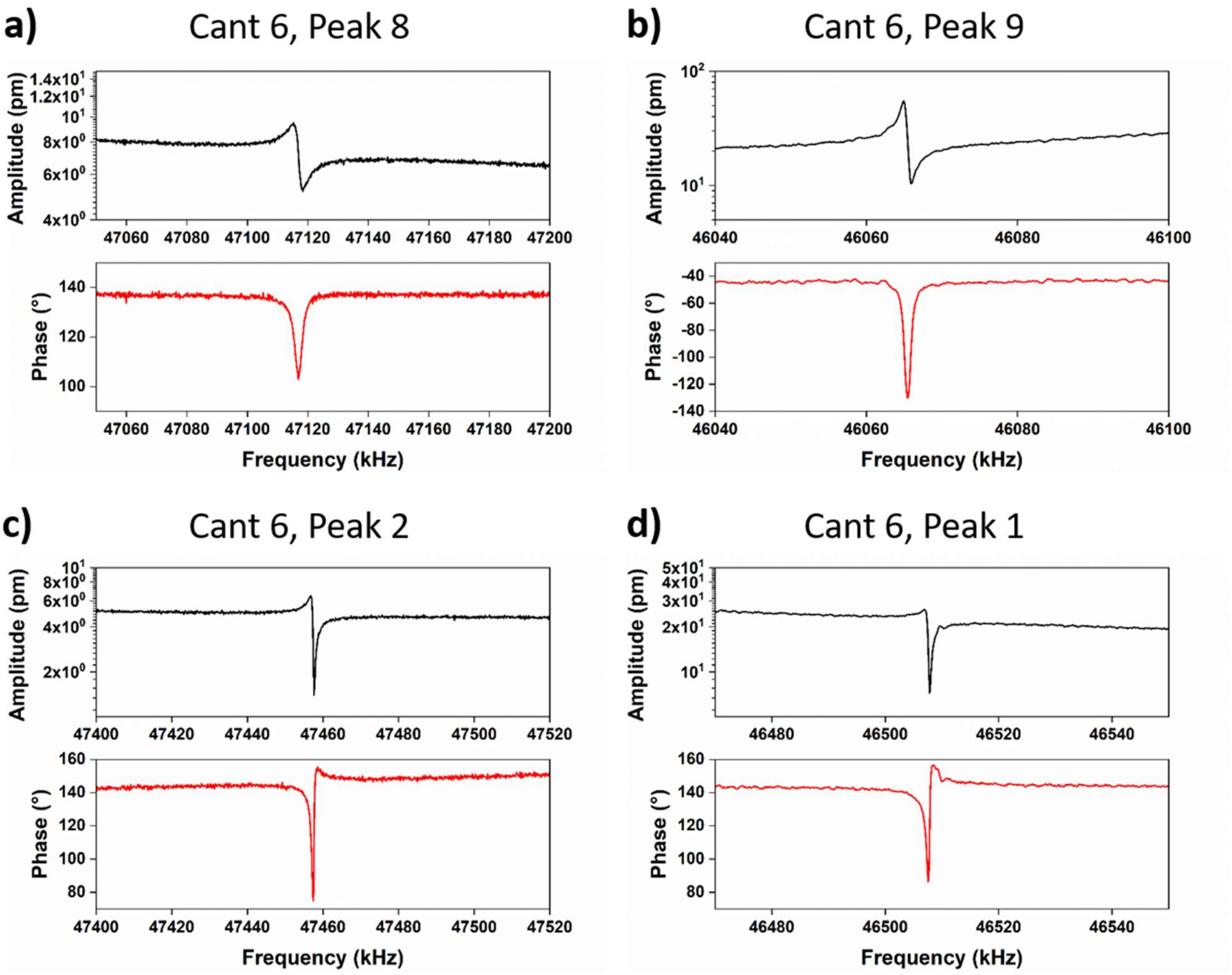

Supplementary Figure 9. Evaluation of phase peak symmetry in another 11-cantilever array. Results analogous to Supplementary Figure 8, but for a different 11-cantilever array. The dimension of the resonators of this array are nominally identical of the one reported in Figure 4 and Supplementary Figure 8. The observed small differences in the resonance frequencies and coupling behavior arise from the fabrication process. See caption of Supplementary Figure 7 for details. Lorentzian peak of Cantilever 6 is located at 46,261 Hz.

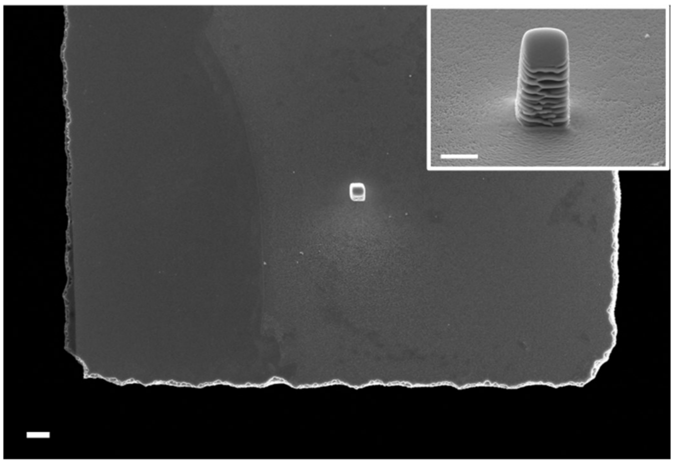

Supplementary Figure 10. Image of the mass deposited on the 44-cantilever array. FESEM images of the right-most cantilever of the resonator array composed by 44 nominally identical cantilevers showing the mass deposited by FIB from a top view and from a view with tilt angle of 54 degree (in the inset). The scale bars are 2 µm in the image and 1 µm in the inset.

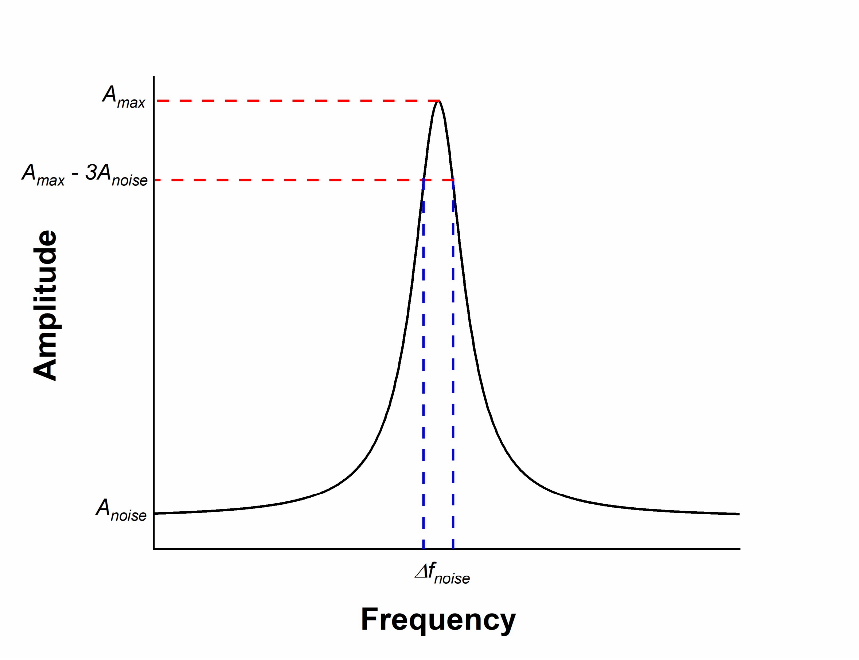

Supplementary Figure 11. Scheme of the Δf_{noise} evaluation. Scheme to compute the SNR and minimum detectable frequency from the *R* signal associated with each weak-coupling peak of the 44-cantilever array. Details of the method to evaluate the minimum detectable frequency are reported in Supplementary Notes 3.

SUPPLEMENTARY NOTES

Supplementary Note 1

Symmetry of the weak-coupling phase peaks. The theoretical model predicts that weak-coupling phenomenon gives rise to the presence of peaks in both the amplitude and phase signals. These amplitude peaks are expected to be asymmetric, with same amplitude above and below the baseline, as shown in Fig. 3b. In contrast, the phase peaks should be symmetric and correspond to a decrease and rise of the phase signal (an inverted peak).

Experimentally both behaviors are not always observed, with the shapes of both the amplitude and phase peaks varying between different fabricated cantilever arrays (of the same nominal structure) and also within resonators of the same device. This suggests that the observed phase asymmetry is driven by non-idealities in fabrication. It is observed that asymmetry in the weak-coupling peak phase signal is present when the maximum and minimum segments of the asymmetric amplitude peak are not antisymmetric; as may be expected. Supplementary Figures 7-9 report examples of the amplitude and phase signals of the weak-coupling peaks recorded on different devices (using the same fabrication process). Resonator arrays with 9 cantilevers or 11 cantilevers are fabricated using the same fabrication process, but their cantilevers have different lengths, widths and distances between neighboring resonators (see Methods). Some resonators display a behavior close to that predicted theoretically, while others show distinct asymmetry in the phase signal. The presence of a symmetric or an asymmetric phase response is independent of the cantilever position in the array (Supplementary Figures 4-5), consistent with fabrication non-idealities.

Supplementary Note 2

Inertial imaging methodology. Inertial imaging is performed using the methodology recently proposed by Sader *et al.*[1] based on multi-mode characterization of the resonator to determine simultaneously the mass, the position and higher-order central moments of the adsorbate mass, such as the variance and the skewness.

This represents a simplified methodology with respect to that proposed originally[2]. The method is based on determination of the central moments of a mass distribution by solving a nonlinear system of equations. This system is derived from the expression for the fractional frequency shifts of the nth mode of a resonator induced by adsorption of mass, *m*:

$$\frac{\delta f_n}{f_n} = -\frac{1}{2M}\int_0^L \mu_{1D}(x)\phi_n^2(x)dx, \tag{1}$$

where M is the resonator mass, $\mu_{1D}(x)$ is the linear mass density of the added particle, L is the resonator length and ϕ_n is the displacement mode shape of the resonator.

Equation (1) can be expressed in terms of added mass *m* and central moments $\langle x^p \rangle$ as:

$$\frac{\delta f_n}{f_n} = -\frac{m}{2M}\left\{\phi_n^2(x_m) + \sum_{p=2}^{\infty} \frac{1}{p!}\frac{d^p\phi_n^2}{dx^p}\bigg|_{x=x_m} \langle x^p \rangle \right\}, \tag{2}$$

From measurements of the fractional frequency shifts of n different modes, solution to the nonlinear system of equation specified by Eq. (1) enables extraction of the added mass *m,* its position x_m and higher-order related central moments, e.g., the variance $<x^2>$, the skewness $<x^3>$ etc. The measurement of n resonance modes allows up to n central moments to be evaluated.

For weakly-coupled cantilever arrays, each of the above-mentioned modes refer to a single CEC of the array.

Supplementary Note 3

Signal-to-noise ratio of the 44-cantilever array. To compute the signal-to-noise Ratio (SNR) of the weak-coupling peaks for an array of 44 cantilevers, we perform a measurement of the amplitude and phase of the vibration spectrum (similar to the one in Fig. 5b) of the cantilever at the border of the array (in this case the left-most). Then we convert the asymmetric shape of the 43 weak-coupling peaks into Lorentzian shape peaks by using the measured amplitude A and phase ϕ signal:

$$X = A\cos\left(\frac{\pi}{180}\phi\right), \tag{3}$$

$$Y = A\sin\left(\frac{\pi}{180}\phi\right), \tag{4}$$

$$R = \sqrt{X^2 + Y^2}. \tag{5}$$

From the results for R, we evaluate the noise level, A_{noise}, and peak amplitude, A_{max}, and then compute the required SNR as:

$$\mathrm{SNR} = \frac{A_{\mathrm{max}}}{A_{\mathrm{noise}}}. \tag{6}$$

The LOD is calculated by estimating the minimum detectable frequency, $\Delta f_{\mathrm{noise}}$, of the (Lorentzian converted) weak-coupling peak as the resonance peak width at amplitude $A_{\mathrm{max}} - 3A_{\mathrm{noise}}$, where A_{max} and A_{noise} are the maximum amplitude of the resonance peak and the noise amplitude, respectively; see Supplementary Figure 11. The theoretical LOD or the minimum detectable mass is obtained as:

$$\mathrm{LOD} = -2M\frac{\Delta f_{\mathrm{noise}}}{f_r}, \tag{7}$$

where M is the mass of the cantilever and f_r is the frequency of the weak-coupling peak.

SUPPLEMENTARY REFERENCES

1 Sader JE, Hanay MS, Neumann AP & Roukes ML Mass Spectrometry Using nanomechanical systems: beyond the point-mass approximation. *Nano Letters* **18**, 1608-1614 (2018).

2 Hanay MS *et al.* Inertial imaging with nanomechanical systems. *Nature Nanotechnology* **10**, 339-344 (2015).
