# Supplementary material for: Large-scale parallelization of nanomechanical mass spectrometry with weakly-coupled resonators
Source: Nat Commun. 2019 Sep 9;10:3647. doi: 10.1038/s41467-019-11647-2 (PMC6733932; doi:10.1038/s41467-019-11647-2)
Supplement: Supplementary file 2 — Peer Review File [file 41467_2019_11647_MOESM2_ESM.docx]

***FIRST ROUND OF REVISIONS***

**Reviewer #2 (Remarks to the Author):**

The Reviewer begins:

*The manuscript entitled Ultimate parallelization of nanomechanical mass spectrometry with weakly coupled resonators presents an elegant method for parallelization of nanomechanical spectrometry based on weak coupling of resonators. The manuscript is very clear and the authors have provided qualitative and quantitative data of the potential of this methods. They have shown this special coupling regime in two different cantilever array configurations and have calculated the Allan variance of the frequencies in order to clearly delineate the ultimate limits of detection. The authors show a proof of concept in which they make “inertial imaging”. I believe that this work is very good and it is a significant advancement. I recommend its publication after correction of some issues,*

*1) The regime of weak coupling deserves a deep description about the mechanism. In which conditions, this phenomenon can be found? The authors claim that there is not overhang, which is the well-known mechanism. This is really an important point. The readers need to know if this is general phenomenon, or something that only can be observed with a very specific conditions.*

We thank the Reviewer for his/her positive assessment and for this suggestion.

In response, a new and comprehensive theory has been developed with new co-authors, Prof. John Sader and Dr Debadi Chakraborty; see “Theoretical model” on Pages 7-11. The implications of this new theory are also explored and compared to measurement. See three new subsections: “Theoretical model”, “Influence of quality factor on weak-coupled peaks” and “Features of the theoretical model” (pages 7-15) and related data/discussion in the Supplementary Information. This replaces the origin and heuristic lumped parameter model with a rigorous treatment of the full 3D elastic problem.

We have also added clarifying comments to “Experimental evaluation of weak-coupling phenomenon”.

These revisions quantify when the weak-coupling peaks can be observed.

The Reviewer’s next comment:

*2) About the description of the state-of-art nanomechanical spectrometry, this is biased to the “school” of inertial imaging or mass spectrometry. The other “school” of naomechanical spectrometry for mass and stiffness detection is somewhat ignored. I recommend to describe both ways to interpret the data in the introduction and justify why the stiffness effect is ignored. In order to balance the introduction, please, include refs. Kosaka et al. (2018, October). Optomechanical devices for deep plasma cancer proteomics. In Seminars in cancer biology (Vol. 52, pp. 26-38). Academic Press, and Ruz, et al (2014). Physics of nanomechanical spectrometry of viruses. Scientific reports, 4, 6051.*

We agree with this (unintentional) lack of balance in the original manuscript, and have revised the manuscript accordingly (page 2):

“This extreme sensitivity has led to the development of nanomechanical mass spectrometry using resonant sensors with applications in the detection of nanoparticles and in proteomics. New theoretical methodologies have also been developed to extract – from the adsorption-induced multimode eigenfrequency variation – an inertial imaging of soft/compliant adsorbates, or the mass, position and stiffness of hard/non-compliant analytes.”

The final request:

*3) In equations 3 and 4 the index m is confusing as it is used for the mass in Eq. (2).*

The new theory removes this issue.

We have also rewritten the manuscript to naturally incorporate the new comprehensive theory.

We trust that these revisions address the Reviewer’s concerns.

**Reviewer #3 (Remarks to the Author):**

The Reviewer’s report:

*The study presents the parallelization of nanomechanical mass spectrometry with weakly coupled resonators, which shows that nanometric resolution of mass and position evaluation of an adsorbed analyte can be evaluated with a single measurement of the frequency response of one resonator. Therefore, the proposed technique using weak coupling peaks is more advantageous as compared to the use of resonance peak in term of analysis time. The manuscript is interesting and certainly deserves publication but in my opinion, it does not match the very high standards of Nature Communications and therefore suggest the manuscript being transferred. Some additional comments:*

*-* ***From a computational method point of view, the approach is extremely simple and I wonder why the authors did not choose a continuum mechanics approach.***

*- Concerning the numerical simulation of the 9 weakly coupled cantilever array: While the mass is changed to fit the experimental data, how are the stiffness and damping factors determined? The authors should also include assumptions and limitations of their simplified model.*

*- It would be interesting to include an uncertainty analysis determining the influence of all uncertain input parameters on the output of interest for both experiments and simulations.*

We thank the Reviewer for his/her comment which has led to extensive revision. The principal criticism lies with use of the (heuristic) lumped parameter model.

We agree and have replaced it with a new rigorous theory, based on an eigenfunction expansion approach, that employs a full 3D treatment of the elastic problem. This is performed in collaboration with new co-authors, Prof. John Sader & Dr Debadi Chakraborty, that allows us to fully explore the mechanisms involved, e.g., we now quantitatively address when the weak-coupling phenomenon can be observed. The new comprehensive theory is presented in the three new subsections: “Theoretical model”, “Influence of quality factor on weak-coupled peaks” and “Features of the theoretical model” (pages 7-15) and related data/discussion in the Supplementary Information.

This new theory now allows us to also explore the influence of uncertainty in the fabrication process on the weak-coupling peaks. See discussion on Page 15 regarding asymmetry in the weak-coupling peaks and the experimental dataset (and discussion) in the Supplementary Information.

The manuscript has been (on the whole) rewritten to naturally include this new theory and discussion.

We trust that this extensive revision addresses the Reviewer’s concern.

***SECOND ROUND OF REVISIONS***

**Reviewer** **#2 (Remarks to the Author):**

The Reviewer states:

*The authors have satisfactorily answered all my questions. The new theoretical model has considerably improved the quality of the manuscript and the potential impact on the readers. I recommend this work for publication in Nature Communications.*

We thank the Reviewer for the positive comments and for the recommendation of publication of the work.

**Reviewer #3 (Remarks to the Author):**

The Reviewer reports:

*This paper demonstrates a parallelization in the detection of extremely small masses with nanoscale resolution via weak coupling. The amplitude and phase response of a single cantilever shows the asymmetric weak coupling peaks originated from other cantilevers in the array. The authors have evaluated the deposited mass and position from the peak shifts for the first 4 collective eigenmode classes of any cantilever in the array. The detailed theoretical framework explores various factors that affect the weak coupling among cantilevers. Furthermore, the limit of the detection for current parallelization and other properties of weak coupling peaks are discussed in detail. The presented scheme seems attractive and can speed-up the analysis process. In summary, I suggest publication and have only a few minor comments/questions:*

1. *Can the current scheme handle a mass deposited on a cantilever at a position less than 0.5 of cantilever length from clamped support?*

We thank the reviewer for the positive comments on the revised manuscript.

The method can handle masses that are in position less than 0.5 of cantilever length. Inertial imaging is based on superposition of the different mode shapes of the resonator (Nature Nanotechnology 10, 339-344 (2015)). The higher the number of modes we are measuring, the higher is the cantilever coverage we can span with the inertial imaging approach. Using the first four resonant modes, we can span from the free end up to 0.2 of cantilever length. Masses deposited in a position less than 0.2 of cantilever length would be difficult to properly evaluate in terms of mass, exact position and dimension, unless we are adding the measurement of higher resonance modes.

2. *Is the deposited mass required to bind strongly to the cantilever? What happens for a loosely bonded deposition?*

Inertial imaging technique works better with small, soft and compliant adsorbates. For such analytes, effects that may arise from adsorbate stiffness can be neglected. Instead, the use of stiff adsorbates can lead to error in the high central moments, e.g., variance and skewness of the adsorbate. For our platinum mass, the variances evaluated with inertial imaging are comparable to those measured using SEM analysis, while some differences exist.

Regarding the reviewer question, we believe that the inertial imaging technique would work properly for loosely bonded masses, unless the masses detach from the surface. Moreover, in case of stiff masses, there could be even a beneficial effect from a loosely bound adsorbate, since the stiffness variation of the cantilever would be limited.

*3. Line 366-382, cantilever labeling seems not appropriate. Weak coupling is recorded for 6th cantilever. Pos1, Pos2, and Pos3 correspond to 7th, 5th and 8th cantilevers from the left, respectively.*

We thank you the reviewer for noticing this error. We made a mistake in labeling the cantilever position in the first revised version of the manuscript. We have now corrected the cantilever position in the text.

3. *The authors are encouraged to mention also related studies as presented e.g. in RSC Advances, 2016, 6, 110466 or Nanoscale, 2014, 6(7), 3618 – 3625*

We have inserted the references suggested by the reviewer in the introduction section, where we mentioned the evolution of nanomechanical sensors.

***THIRD ROUND OF REVISIONS***

**Reviewer #3 (Remarks to the Author):**

The Reviewer states:

*The manuscript has been convincingly revised and I therefore suggest publication in its present form.*

We thank the Reviewer for the positive comment and for the recommendation of publication of the work.
